# Sulfur Isotopes Reveal Spatial Variation in Waterbird Trace Element Contamination from Tropical Estuaries to the Open Ocean

**DOI:** 10.1021/acs.est.5c14633

**Published:** 2026-03-19

**Authors:** Bruno A. Linhares, Paco Bustamante, Guilherme T. Nunes, Patrícia G. Costa, Leandro Bugoni, Yuri D. Zebral, Iole B. M. Orselli, Camila M. G. Martins, Adalto Bianchini

**Affiliations:** † Programa de Pós-Graduação em Oceanografia Biológica, Instituto de Oceanografia, 67820 Universidade Federal do Rio Grande − Furg, Rio Grande, Rio Grande do Sul 96203-900, Brazil; ‡ 27018 Littoral Environnement et Sociétés (LIENSs), UMR 7266 CNRS - La Rochelle Université, La Rochelle 17000, France; § Institut Universitaire de France (IUF), 1 rue Descartes, Paris 75005, France; ∥ Centro de Estudos Costeiros, Limnológicos e Marinhos, Universidade Federal do Rio Grande do Sul − UFRGS, Imbé, Rio Grande do Sul 95625-000, Brazil; ⊥ Laboratório de Determinações 2, Instituto de Ciências Biológicas, Universidade Federal do Rio Grande − FURG, Rio Grande, Rio Grande do Sul 96203-900, Brazil; # Laboratório de Aves Aquáticas e Tartarugas Marinhas, Instituto de Ciências Biológicas, Universidade Federal do Rio Grande − FURG, Rio Grande, Rio Grande do Sul 96203-900, Brazil; ∇ Programa de Pós-Graduação em Ciências Fisiológicas, Instituto de Ciências Biológicas, Universidade Federal do Rio Grande − FURG, Rio Grande, Rio Grande do Sul 96203-900, Brazil

**Keywords:** arsenic, blood, cadmium, ecotoxicology, lead, mercury, seabirds, stable isotope analysis

## Abstract

Marine and freshwater pollution is a major environmental concern, yet the spatial extent of estuarine contamination in marine food webs remains poorly understood. In this study, we sampled blood from piscivorous waterbirds (15 species, 316 individuals) along a gradient from estuaries in southeastern Brazil to the nearshore and offshore southwestern Atlantic Ocean to assess trace element concentrations and the influence of habitat and trophic position on the basis of stable isotopes of carbon, nitrogen, and sulfur. All nonessential trace elements (arsenic, cadmium, lead, and mercury) were inversely related to sulfur isotope values (δ^34^S) and decreased along the estuarine–open ocean gradient. Conversely, most essential elements (chromium, copper, and manganese) were positively correlated with δ^34^S, suggesting an increase from estuarine to oceanic waters. Nonessential trace elements in estuarine birds were above the benchmarks of toxicity established for bird species, suggesting potential health impairment due to trace element contamination associated with freshwater inputs. In addition, the lower concentrations of essential elements in estuarine birds may suggest interference in metal homeostasis caused by high concentrations of nonessential elements. Assessments combining multiple trace elements and stable isotopes, particularly sulfur, remain rare over large spatial scales. Across this broad environmental gradient, stable sulfur isotopes proved to be efficient markers of bird habitats for assessing spatial patterns in trace element contamination in aquatic food webs.

## Graphical abstract

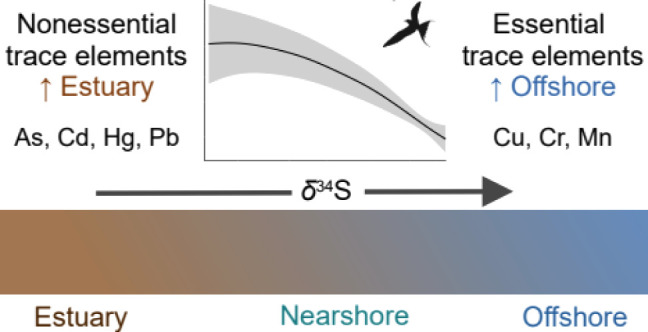

## 1 Introduction

The increased concentrations of potentially toxic trace elements in aquatic environments pose risks to human and environmental health because of their potential adverse effects.^1^ Nonessential trace elements such as arsenic (As), cadmium (Cd), mercury (Hg), and lead (Pb) are toxic even at low concentrations,^2^ but the amount of these elements circulating in ecosystems continues to increase because of industrial, agricultural and mining activities.^3–5^ By contrast, essential trace elements such as chromium (Cr), copper (Cu), iron (Fe), manganese (Mn) and zinc (Zn) are required for metabolism and physiologically regulated, but health impairment can occur if their concentrations are too low or too high, leading to deficiency or toxicity, respectively.^6,7^ Nonessential trace elements may also interfere with essential metal regulation, potentially expanding physiological effects.^7,8^ Trace metals from both natural (e.g., erosion of rocks and soils) and anthropogenic sources may be transported to the ocean by river runoff and atmospheric deposition.^9,10^ While atmospheric transport contributes to the concentrations of some elements in the open ocean (e.g., Hg and Pb), trace elements transported by river runoff may be scavenged by suspended particles during estuarine mixing and retained in coastal sediments.^9^ This retention is more pronounced for particle-reactive elements (e.g., As, Hg, Pb, and Zn), which readily associate with organic matter and Fe–Mn oxyhydroxides.^9,11–13^ Under changing environmental conditions, sediments may act as secondary sources of trace metals to the water column due to the remobilization of particle-associated metals.^12,14^ The feeding habits of organisms, such as foraging habitat, diet composition and trophic level, also influence contaminant exposure.^15–17^ For instance, Hg is known to biomagnify in food webs in its organic form (methylmercury, MeHg),^18,19^ whereas other nonessential trace elements (e.g., Cd and Pb) tend to decrease across trophic levels.^20–22^ Discerning the biogeochemical factors that influence trace element contamination in aquatic food webs has been a long-standing challenge for human and environmental safety, highlighted in international regulatory efforts targeting specific contaminants such as Hg (e.g., the Minamata Convention).

Bioindicator organisms can be used to assess and monitor trace element contamination in aquatic ecosystems. Aquatic predators are proxies of food web contamination, as they are exposed to contaminants assimilated by lower trophic levels, making them powerful tools for assessing environmental contamination.^17,23^ In this sense, fish-eating birds have been historically used as indicators of aquatic pollution,^24,25^ because they (i) occupy a range of ecosystems, including the ocean (i.e., seabirds); (ii) feed on high trophic levels, potentially sharing resources with humans (e.g., commercial fishes); (iii) have wide foraging areas but can be sampled in local aggregations, such as in breeding colonies; and (iv) facilitate the collection of samples nondestructively, such as blood and feathers, allowing less invasive monitoring of contaminant levels.^26^ The circulating blood is generally used for assessing recent exposure to contaminants (days to weeks), although mobilization from internal organs and physiological regulation—especially for essential elements—may also alter blood metal concentrations.^24,25,27,28^ Consequently, trace element concentrations in bird blood are often associated with tracers of its recent trophic ecology, such as stable isotopes also analyzed in blood to assess foraging areas and diet.^15,16,23,26,29^

Stable isotopes have been used as biogeochemical tracers of the feeding ecology of predators for decades.^30^ Carbon (δ^13^C) and nitrogen (δ^15^N) stable isotopes are the most commonly used to assess the feeding habitats and trophic positions, respectively. There is an increase in δ^15^N at each trophic transfer by ∼3.4 ‰ and therefore it has been widely used as a proxy for trophic position.^31^ However, the utility of bulk δ^15^N as trophic position indicator may be undermined by spatial and temporal differences in baseline δ^15^N values.^31^ For instance, coastal waters often exhibit higher δ^15^N baselines than offshore waters due to increased organic matter inputs and microbial processing, which lead to overall ^15^N enrichment.^32,33^ In contrast, carbon isotope values vary by <1 ‰ across trophic levels and roughly reflect carbon assimilation by producers during photosynthesis, making δ^13^C a useful tracer of feeding habitats.^34^ Offshore waters generally exhibit lower δ^13^C baselines than coastal waters due to lower primary productivity and higher CO_2_ availability, favoring fractionation against the heavier ^13^C isotope.^30,33^ Estuarine zones may also present lower δ^13^C values than coastal waters due to inputs of ^13^C-depleted organic matter from continental C_3_ plants.^35^ Consequently, δ^13^C values of primary producers can overlap across estuarine, coastal, and oceanic systems, confounding its use as a precise feeding habitat tracer across these habitat boundaries.^33,36^ Therefore, additional biogeochemical tracers have increasingly been used to assess the feeding habitats of consumers across coastal systems.^17,36,37^ For instance, the sulfur isotopic values (δ^34^S) assimilated from environmental sulfates are minimally altered by organisms across trophic levels.^38,39^ Sulfate in seawater is abundant and enriched in ^34^S, whereas freshwater systems typically exhibit lower δ^34^S values due to inputs of organic matter derived from aquatic and terrestrial plants utilizing ^34^S-depleted sulfides.^40^ Sulfur isotopes have proven useful for assessing migratory movements and contaminant exposure in fishes,^37^ sharks,^17^ and seabirds across coastal (lower δ^34^S) and oceanic waters (higher δ^34^S).^23,29,36^ Despite the importance of predator habitat discrimination in contaminant studies, combined assessments of trace elements and sulfur, carbon and nitrogen isotopes across multiple species and ecosystems remain rare. This underscores the need to evaluate isotopic and contaminant variability across broad environmental gradients, such as from estuaries to the open ocean.

Important areas for investigating the dispersion of coastal pollutants are those that have been impacted by known pollution sources. In southeastern Brazil, trace elements have been released into rivers through the mining of gold (Au), Fe, and Mn since the 17th Century, in addition to inputs from urban, industrial and agricultural development.^14^ Allegedly, the largest mine-dam accident worldwide occurred in the region in 2015, when an Fe mining dam (Fundão Dam) collapsed, releasing approximately 50 million cubic meters of toxic ore tailings into the socioenvironmentally important Doce River Basin.^14^ Tailings containing high concentrations of multiple trace elements were transported more than 600 km, where they reached the estuary, adjacent rivers and the southwestern Atlantic Ocean.^41,42^ Following the initial impact, resuspension of deposited tailings during periods of severe rainfall, river runoff, and marine cold fronts has contributed to ongoing trace element inputs to aquatic systems.^12,43–45^ Recent studies have reported concentrations of trace elements above reference values for toxic impacts in the blood and feathers of marine and estuarine birds sampled in the impacted areas.^26,46,47^ These studies suggest that trace element concentrations in bird tissues are influenced by climate-driven inputs, as well as by the foraging habitats and diet composition of seabirds, as inferred from stable isotopes and tracking data.^16,26,46,47^ Nonetheless, there is still a gap regarding the spatial extent of trace element contamination in marine food webs in the region. In this sense, piscivorous birds constitute a widespread ecological guild of aquatic predators that can be sampled across estuarine and marine habitats, thereby providing a suitable framework for assessing spatial variation in coastal pollution. Assessing trace element exposure in aquatic predators across coastal gradients, from impacted estuaries to the open ocean, may be key for coastal impact assessments at regional and global scales.

To assess spatial variation in coastal pollution from estuaries in southeast Brazil to the nearshore and offshore southwestern Atlantic Ocean, we used piscivorous waterbirds as biomonitors. Specifically, we (i) use stable isotopes of carbon, nitrogen, and sulfur to characterize foraging habitats and ecological structure of waterbird assemblages across estuarine, nearshore, and offshore habitats; (ii) examine how these patterns relate to broad-scale spatial variation in a suite of essential (Cr, Cu, Fe, Mn, and Zn) and nonessential (As, Cd, Hg, and Pb) trace elements measured in bird blood; and (iii) evaluate habitat-specific relationships between stable isotopes and trace element concentrations across waterbird assemblages. We expected trace element concentrations to decrease from estuarine to offshore habitats in response to decreasing continental influence. We predicted that sulfur isotopes would provide the strongest spatial resolution among waterbird habitats, thereby explaining variation in trace element exposure across the coastal gradient.^36^ In addition, we expected positive relationships between δ^15^N values and Hg concentrations due to the biomagnification of MeHg.^19^ Overall, this study aims to contribute to the understanding of broad-scale spatial variability in trace element contamination on tropical coasts and the use of piscivorous waterbirds as model organisms to assess contamination patterns across ecological scales.

## 2 Materials and Methods

### 2.1 Study Area and Sample Collection

Blood samples (*n* = 316) from 15 species of piscivorous waterbirds were collected across estuarine, nearshore and offshore habitats in southeastern Brazil and the southwestern Atlantic Ocean between 2007 and 2024. Estuarine samples were collected in the Doce River estuary and in two nearby estuaries, while marine samples were collected from seabirds in the nearshore Abrolhos Archipelago (ca. 70 km from the coast) and on the offshore Trindade Island (ca. 1,200 km offshore) (Table 1 and Figure 1). To characterize habitat-level contamination while accounting for interspecific variability in trophic ecology and contaminant exposure,^16^ we sampled at least five species per habitat. Because sampling occurred across multiple years and simultaneous sampling of all species/habitats was not feasible, multiple sampling events per species were included to ensure adequate representation across species and sampling periods, while accounting for temporal variability in contaminant exposure documented in previous studies.^26,46,47^ Sampling procedures were conducted under permits issued by Brazilian environmental authorities (SISBIO 64261-13 and 64381) and approved by the institutional animal ethics committee (CEUA-FURG 23116.001336/2020-40 and 23116.003357/2023-42). Additional fieldwork details are available in the Supporting Information and elsewhere.^26,46,47^

**Table 1:** Summary Information of Piscivorous Waterbird Assemblages Sampled across Estuaries, Nearshore and Offshore Habitats in Brazil, Including Sampling Locations, Species and the Number of Blood Samples (n) Collected by Year and Species^a^

Environment (*n*): Location (lat, long)	Sampling year (*n*)	Common name (Scientific name,*n*)	Variable	Mean ± SD	95% CI	Median
			δ^13^C	–20.43 ± 3.31	(−21.07, −19.78)	–19.59 a
			δ^15^N	11.57 ± 2.25	(11.13, 12.01)	12.09 a
**Estuary** (*n* = 106):			δ^34^S	13.34 ± 4.85	(12.41, 14.27)	13.01 a
	2018 (*n* = 3);	Green kingfisher (*Chloroceryle americana*, *n* = 16);	As	1.05 ± 2.07	(0.65, 1.45)	0.33 a
Doce	2019 (*n* = 17);	Amazon kingfisher (*C. amazona*, *n* = 29);	Cd	0.31 ± 0.51	(0.21, 0.41)	0.10 a
(−19.63°S, −39.82°W);	2020 (*n* = 3);	Ringed kingfisher (*Megaceryle torquata*, *n* = 9);	Hg	0.62 ± 1.85	(0.26, 0.98)	0.05 a
São Mateus	2021 (*n* = 9);	Black skimmer (*Rynchops niger*, *n* = 16);	Pb	0.24 ± 0.38	(0.17, 0.32)	0.07 a
(−18.60°S, −39.74°W);	2022 (*n* = 28);	Common tern (*Sterna hirundo*, *n* = 19);	Cr	1.84 ± 3.38	(1.19, 2.5)	0.63 a
Piraquê-açu	2023 (*n* = 24);	Cabot’s tern (*Thalasseus acuflavidus*, *n* = 17)	Cu	1.57 ± 4.61	(0.66, 2.48)	0.39 a
(−19.95°S, −40.16°W)	2024 (*n* = 22).		Fe	160.26 ± 603.67	(44.0, 276.52)	14.89 a
			Mn	5.69 ± 16	(2.53, 8.85)	1.55 a
			Zn	10.16 ± 27.1	(4.94, 15.37)	3.21 a
—	—	—	—	—	—	—
			δ^13^C	–17.78 ± 0.90	(−17.93, −17.62)	–17.76 b
		Brown booby (*Sula leucogaster*, *n* = 27);	δ^15^N	10.93 ± 1.60	(10.65, 11.21)	10.69 b
**Nearshore** (*n* = 125)	2011 (*n* = 31);	Masked booby (*S. dactylatra*, *n* = 23);	δ^34^S	19.89 ± 1.09	(19.7, 20.09)	20.29 b
	2022 (*n* = 30);	Brown noddy (*Anous stolidus*, *n* = 24);	As	0.42 ± 0.44	(0.34, 0.49)	0.34 a
Abrolhos Archipelago	2023 (*n* = 18);	Magnificent frigatebird (*Fregata magnificens*, *n* = 28);	Cd	0.03 ± 0.06	(0.02, 0.04)	0.02 b
(−17.97°S, −38.7°W)	2024 (*n* = 46)	Red-billed tropicbird (*Phaethon aethereus*, *n* = 23)	Hg	0.11 ± 0.10	(0.1, 0.13)	0.09 a,b
			Pb	0.08 ± 0.15	(0.05, 0.10)	0.03 b
			Cr	3.4 ± 4.41	(2.61, 4.18)	0.61 a
			Cu	4.9 ± 9.61	(3.18, 6.62)	2.15 b
			Fe	7.57 ± 8.65	(6.04, 9.10)	5.10 b
			Mn	6.37 ± 11.19	(4.37, 8.37)	1.51 a
			Zn	13.82 ± 23.57	(9.64, 17.99)	3.47 a
—	—	—	—	—	—	—
			δ^13^C	–17.82 ± 0.86	(−18.01, −17.64)	–17.54 b
			δ^15^N	9.2 ± 1.37	(8.9, 9.49)	8.80 c
			δ^34^S	20.72 ± 0.42	(20.63, 20.81)	20.75 c
			As	0.43 ± 0.31	(0.37, 0.50)	0.46 a
		Brown noddy (*Anous stolidus*, *n* = 11);	Cd	0.01 ± 0.01	(0.01, 0.01)	0.01 c
**Offshore** (*n* = 85):	2007 (*n* = 31);	White tern (*Gygis alba*, *n* = 16);	Hg	0.17 ± 0.31	(0.11, 0.24)	0.16 b
	2017 (*n* = 13);	Sooty tern (*Onychoprion fuscatus*, *n* = 19);	Pb	0.04 ± 0.05	(0.03, 0.05)	0.03 b
Trindade Island	2022 (*n* = 41)	Masked booby (*Sula dactylatra*, *n* = 19);	Cr	6.9 ± 4.65	(5.89, 7.9)	9.31 b
(−20.51°S, −29.33°W)		Trindade petrel (*Pterodroma arminjoniana*, *n* = 20)	Cu	3.05 ± 5.9	(1.7, 4.4)	0.58 c
			Fe	7.91 ± 7.34	(6.33, 9.49)	6.37 b
			Mn	11.24 ± 10.28	(9.02, 13.45)	10.67 b
			Zn	12.77 ± 10.95	(10.41, 15.13)	11.50 b

a For each habitat, the mean ± standard deviation, median and the 95% confidence interval of the mean (CI) are reported for trace element concentrations (mg/L ww) and stable isotope values (‰) (see Table S1 for values by species). For each element, letters following the median indicate results of pairwise comparisons across habitats (species pooled), where different letters denote significant difference between habitats (*p* < 0.05).

**Figure 1:**
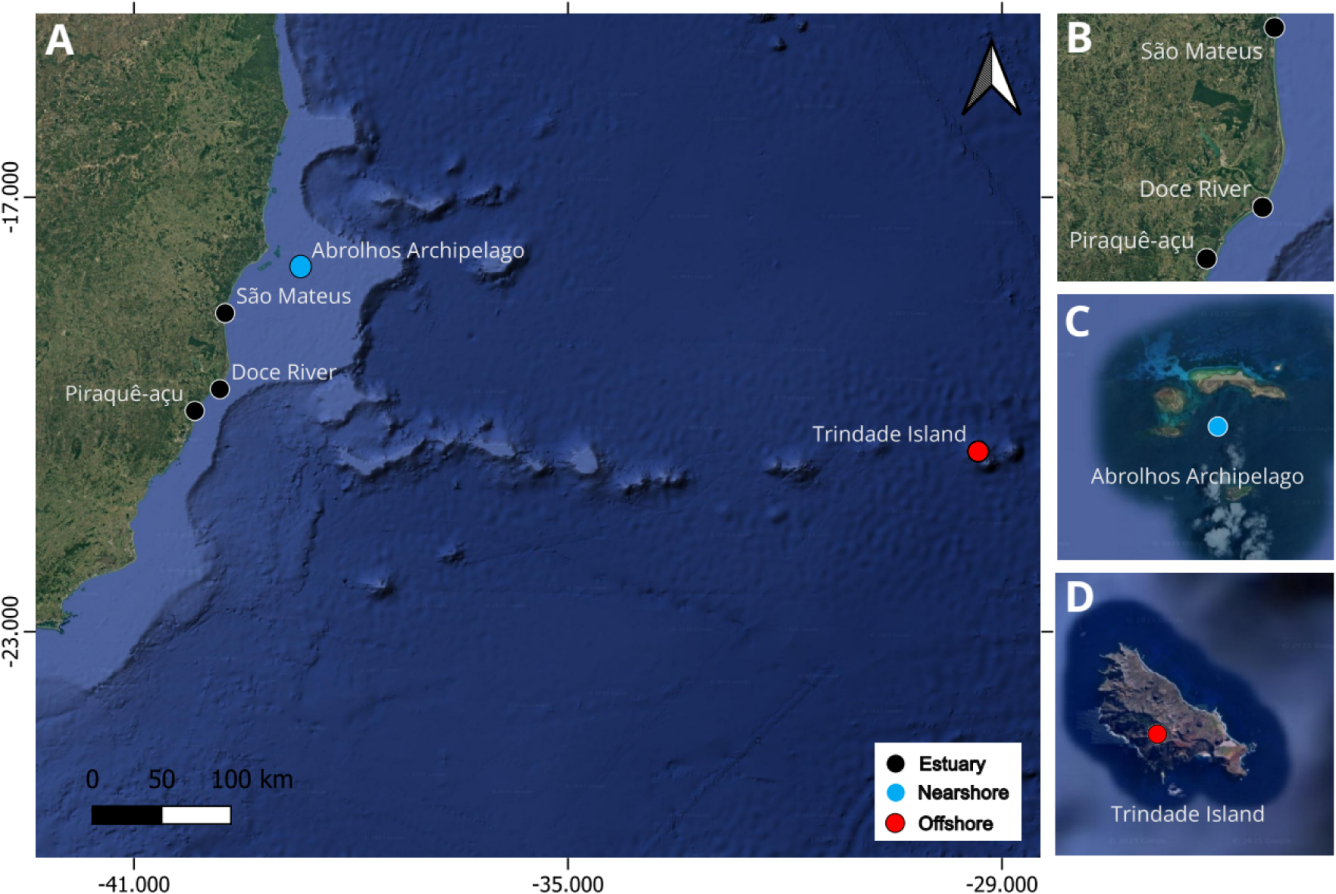
Sampling areas in southeastern Brazil and the southwestern Atlantic Ocean (A), with a magnified view of the estuaries (B), nearshore (C; Abrolhos Archipelago) and offshore (D; Trindade Island) marine habitats, where blood samples of piscivorous waterbirds were collected.

### 2.2 Laboratory Analyses

Freeze-dried whole blood samples were analyzed for δ^13^C and δ^15^N at the Centro Integrado de Análises (CIA-FURG, Brazil) and for δ^34^S at La Rochelle Université (LIENSs, France), using an elemental analyzer coupled with an isotope ratio mass spectrometer (IRMS, Thermo Scientific). Sample isotopic values (δ) are expressed in per mil (‰) in relation to international standards. Analyses of internal laboratory standards (caffeine, acetanilide and glutamic acid for δ^13^C and δ^15^N and USGS42 and IAEA-S-2 for δ^34^S) interspersed between samples yielded measurement accuracies of 0.1 ‰, 0.3 ‰ and 0.1 ‰ for carbon, nitrogen and sulfur stable isotopes, respectively. For trace element analysis, dry blood samples were digested using 65% ultrapure nitric acid (HNO_3_) in a microwave preparation system. Digested samples were diluted with high-purity deionized water. Concentrations were determined on the basis of calibration curves constructed for each metal using a serial dilution prepared from a multielement standard solution (1000 mg/L). Samples were processed using standardized laboratory protocols, using inductively coupled plasma mass spectrometry (ICP-MS, PlasmaQuant MS Q, Analytik Jena) or high-resolution continuum source graphite furnace atomic absorption spectrometry (HR-CS GF AAS, Analytik Jena), with Hg quantified by atomic fluorescence spectrometry (Mercur Duo Plus). Quality control and assurance were based on the regular analysis of blanks, spiked matrices and certified reference materials (e.g., TORT-3, lobster hepatopancreas; DOLT-5 and DORM-4, dogfish liver and muscle, respectively; National Research Council, Canada), with analytical accuracy and precision within acceptable limits. The mean recovery rates were between 81% for Zn and 88% for Hg. The limits of detection (LOD) and quantification (LOQ) were three and ten times the blank signals, respectively. For a small number of samples (detection rates >0.8 for all elements), values below the LOQ were replaced by LOQ/2.

### 2.3 Statistical Analyses

Statistical analyses were conducted in R 4.3.1.^48^ Factorial differences in stable isotopes and trace elements across habitats (pooled species) were tested with Kruskal-Wallis and the post hoc Dunn’s test.^49^ The Pearson correlation was computed prior to conducting a principal component analysis (PCA) in *FactoMineR*,^50^ using scaled values of stable isotopes and trace elements (mg/L ww; log-transformed) due to different magnitudes and units across variables. Generalized additive models (GAMs) were implemented with the *mgcv* package to assess potential nonlinear relationships between stable isotope values and log-transformed concentrations of nonessential trace elements (As, Cd,Hg and Pb).^51^ Full models included all stable isotopes as explanatory smooth terms and accounted for species-specific temporal variation by incorporating a factor-smooth interaction between the species and the sampling year (with 0.5 added for the winter season). Models were fitted using restricted maximum likelihood (REML), which provides stable estimations of smoothing parameters when modeling potentially nonlinear and correlated predictors. Variable selection was performed within full GAMs by enabling shrinkage penalization as implemented in *mgcv*.^51^ In addition, GAMs including only stable isotopes as explanatory variables were fitted to evaluate the explanatory power of isotopic predictors without accounting for species-specific temporal trends.

## 3 Results

Sulfur isotope values revealed a clear gradient across habitats, with lower values in the estuarine bird assemblage (mean ± SD = 13.34 ± 4.85 ‰), increasing in nearshore (19.89 ± 1.09 ‰) and offshore bird assemblages (20.72 ± 0.42 ‰) (Figure 2; Kruskal-Wallis χ^2^ = 197.02, *p* < 0.001). All δ^34^S pairwise comparisons between habitats were significant (Dunn’s post hoc test in Table 1 and Figure 2). Sulfur isotope values were positively correlated with carbon (*r* = 0.52, *p* < 0.001) and negatively correlated with nitrogen isotope values (*r* = −0.22, *p* < 0.001) (Figure S1). Carbon isotopic values showed greater similarity among habitats than sulfur isotopes (Figure 2), and δ^13^C values of the nearshore and offshore bird assemblages did not differ significantly (Table 1). Estuarine birds presented the lowest δ^13^C values among habitats (χ^2^ = 70.56, *p* < 0.001); however, the dispersion of δ^13^C values across estuarine species (except the green kingfisher *Chloroceryle americana*) largely fell within the range observed in offshore and nearshore assemblages (Table S1; Figures 2 and S2). Notably, some of the highest δ^13^C values were observed in the estuary (Figure 2), including the highest individual value recorded (Amazon kingfisher *C. amazona*, −11.8 ‰). Finally, δ^15^N values differed across habitats (χ^2^ = 72.00, *p* < 0.001), with the highest values in the estuary (11.57 ± 2.25 ‰) and the lowest in the offshore habitat (9.2 ± 1.37 ‰), with all pairwise comparisons significant (Table 1).

**Figure 2:**
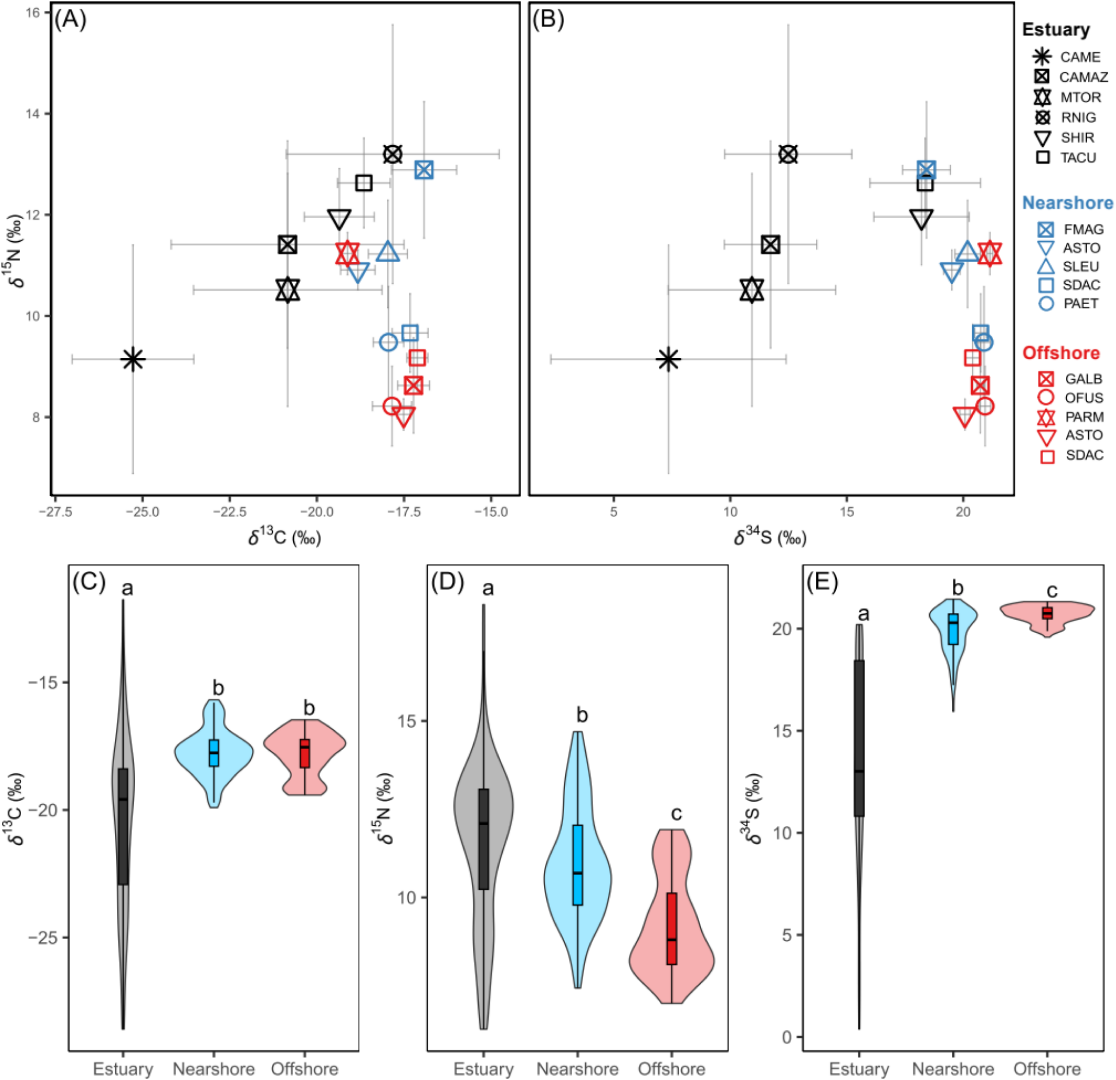
Stable isotope biplots (above) with the mean ± standard deviation of each piscivorous bird species (symbols) sampled in estuarine (black), nearshore (blue) and offshore (red) habitats in Brazil, representing δ^13^C vs δ^15^N (A) and δ^34^S vs δ^15^N (B). Panels below (C–E) show habitat-level distributions of δ^13^C, δ^15^N, and δ^34^S values, respectively. Boxes indicate medians and interquartile ranges, while violins represent data density. Different letters denote significant pairwise differences among habitats based on Dunn’s post hoc tests (*p* < 0.05). Abbreviations: CAME = *Chloroceryle americana*; CAMAZ = *C. amazona*; MTOR = *Megaceryle torquata*; RNIG = *Rynchops niger*; SHIR = *Sterna hirundo*; TACU = *Thalasseus acuflavidus*; ASTO = *Anous stolidus*; PAET = *Phaethon aethereus*; SDAC = *Sula dactylatra*; SLEU = *S. leucogaster*; FMAG = *Fregata magnificens*; OFUS = *Onychoprion fuscatus*; GALB = *Gygis alba*; and PARM = *Pterodroma arminjoniana*.

Among nonessential trace elements, Cd and Pb concentrations were higher in the estuary than in the nearshore and offshore habitats (χ^2^ = 121.5, *p* < 0.001 and χ^2^ = 23.41, *p* < 0.001, respectively), and Cd was also higher nearshore than offshore (Table 1). Mercury differed significantly among habitats (χ^2^ = 12.99, *p* = 0.002), with higher concentrations offshore than in the estuary (Table 1). Arsenic did not differ significantly among habitats (χ^2^ = 1.31, *p* = 0.519). Regarding the essential trace elements, Fe was highest in the estuary (χ^2^ = 27.21, *p* < 0.001), while Cr, Mn, and Zn were highest offshore. Copper was highest nearshore and lowest in the estuary (χ^2^ = 45.45, *p* < 0.001; Table 1).

The PCA revealed clear distinctions between estuarine and marine species along PC2, which explained 22.2% of the total variation. PC2 was negatively associated with δ^34^S (*r* = −0.75, contribution = 21.3%) and δ^13^C (*r* = −0.73, contribution = 19.9%), and positively associated with Cd (*r* = 0.77, contribution = 22.4%), Fe (*r* = 0.49, contribution = 9.2%) and Pb (*r* = 0.67, contribution = 17.1%) (Figure 3). Chromium, Cu and Mn were positively correlated with δ^34^S (Figure S1) and negatively correlated with PC2 (*r* = −0.22, *r* = −0.26 and *r* = −0.21, respectively) (Figure 3). PC1 explained 32.5% of the total variation and showed weak associations with stable isotope values and spatial structure. PC1 is characterized by positive correlations among As, Cr, Cu, Mn, Hg and Zn, with individual contributions ranging from 9.4 to 18.2%. Nitrogen isotope values exhibited low squared cosines (cos^2^ = 0.08), indicating limited representation in the first two principal components (Figure 3).

**Figure 3:**
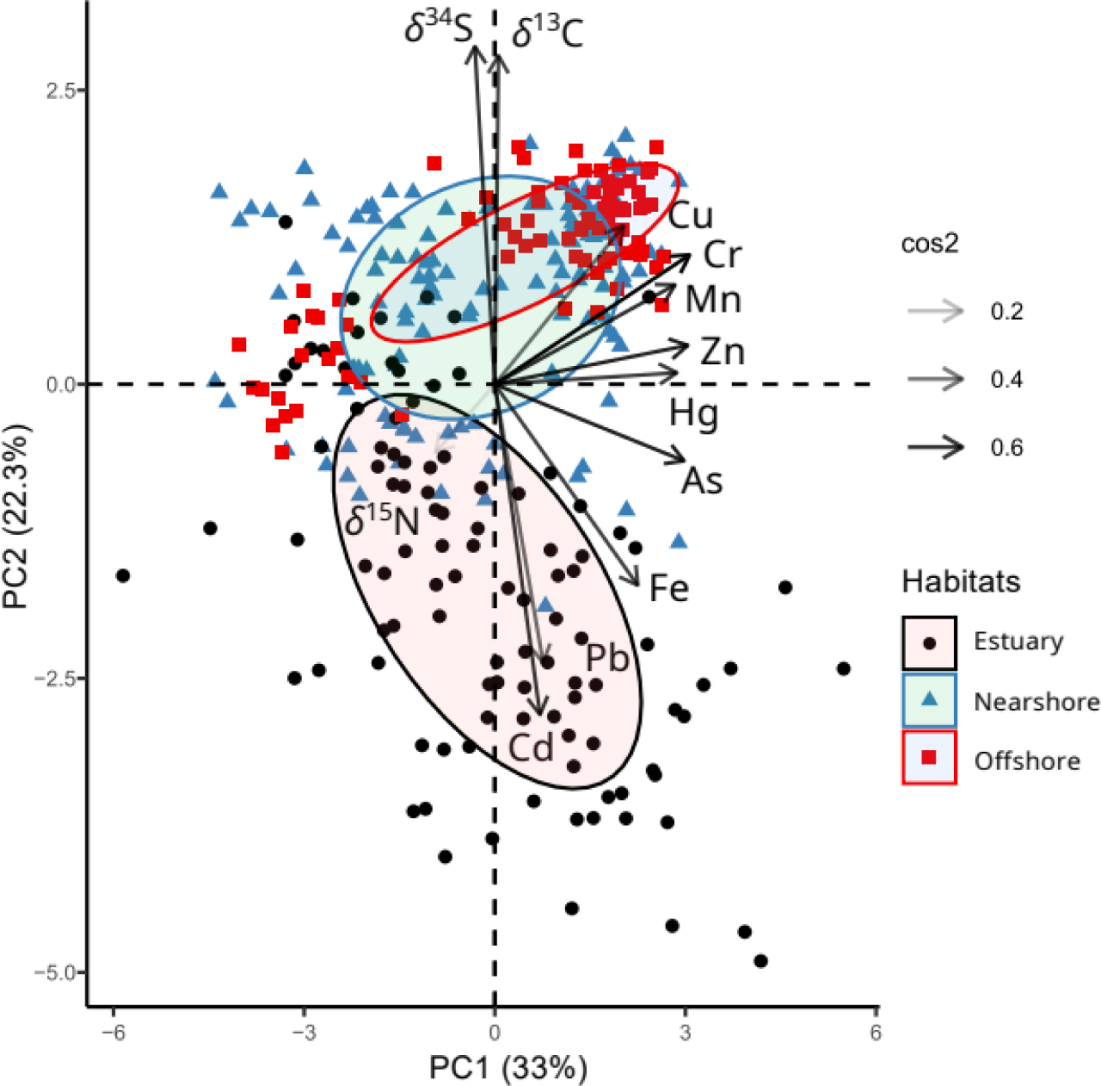
Principal component analysis (PCA) of the scaled concentrations of trace elements (log-transformed) and stable isotope values in piscivorous waterbirds sampled across estuarine, nearshore and offshore habitats. Arrow colors represent the proportion of variance of each variable explained by principal components (cos^2^).

Sulfur isotopes and species-specific temporal structure were retained as significant explanatory variables in all full GAMs fitted for nonessential trace elements (*p* < 0.05; Table S2). Overall, nonessential trace element concentrations decreased with increasing δ^34^S values (Figure 4). Carbon isotopes were significant in the Cd model (*F* = 1.78, *p* < 0.001), indicating a nonlinear relationship (Figure S3). Nitrogen isotopes were significant in the Cd (*F* = 1.16, *p* = 0.001), Hg (*F* = 2.39, *p* = 0.03) and Pb (*F* = 1.38, *p* < 0.001) models, showing overall negative relationships with these trace elements (Table S2 and Figure S4). Deviance explained by full models versus isotope-only models was 44.2% and 17.7% for As (Δ = 26.5%), 55.4% and 37.3% for Cd (Δ = 18.1%), 58.7% and 14.0% for Hg (Δ = 44.7%), and 45.4% and 17.9% for Pb (Δ = 27.5%). In the estuarine data set, δ^15^N values showed negative relationships with Cd, Hg and Pb (Table S3 and Figure 5). In the nearshore habitat, As, Hg, and Pb decreased with increasing δ^34^S values, Pb decreased with increasing δ^15^N values, and Cd and Hg increased with increasing δ^15^N values (Table S4 and Figure 5). In the offshore habitat, δ^34^S values were significantly associated with As and Pb (Table S4 and Figure S5).

**Figure 4:**
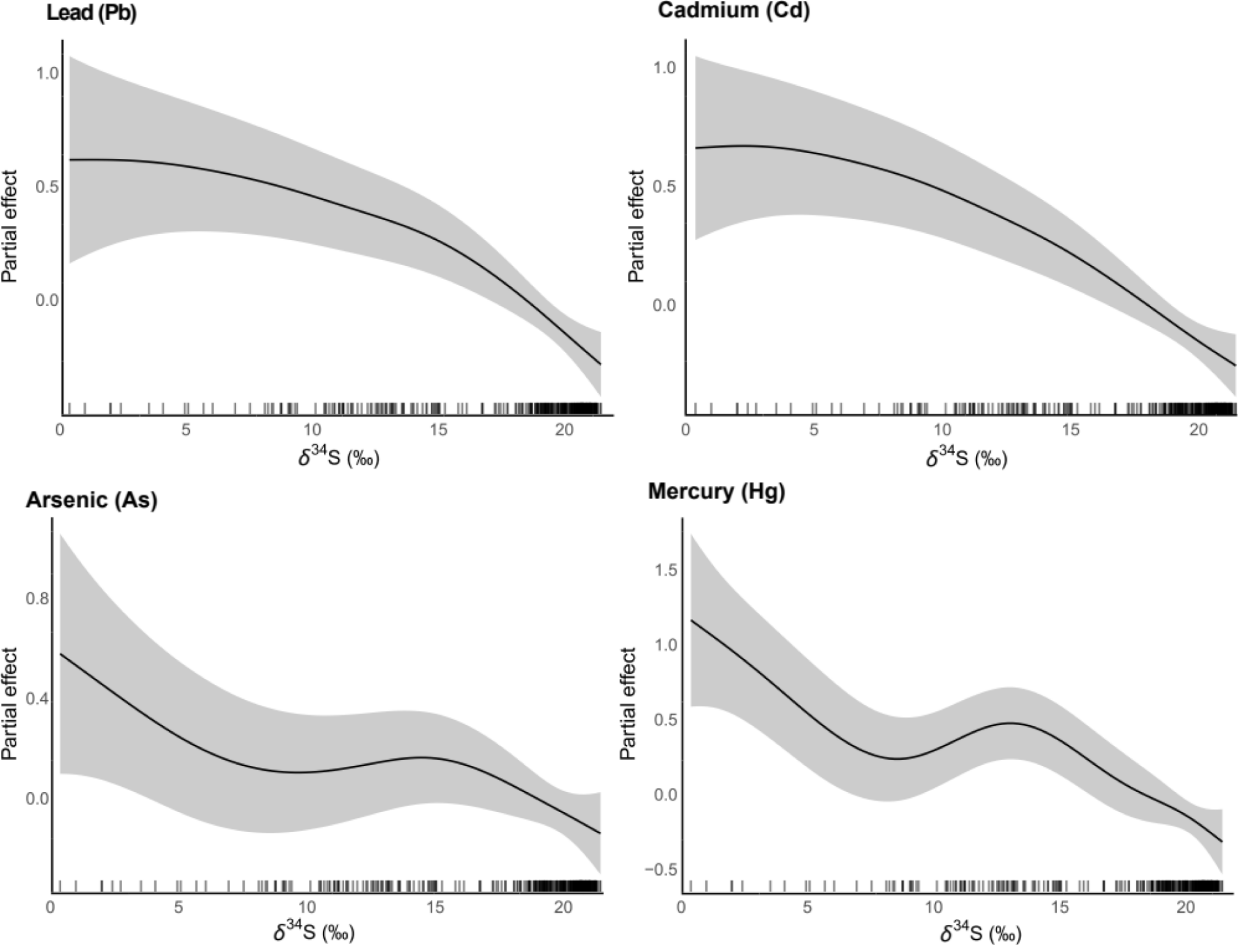
Smooths obtained with generalized additive models, representing the partial effect of δ^34^S over nonessential trace elements measured in the blood of waterbirds sampled in estuarine, nearshore and offshore habitats in Brazil.

**Figure 5:**
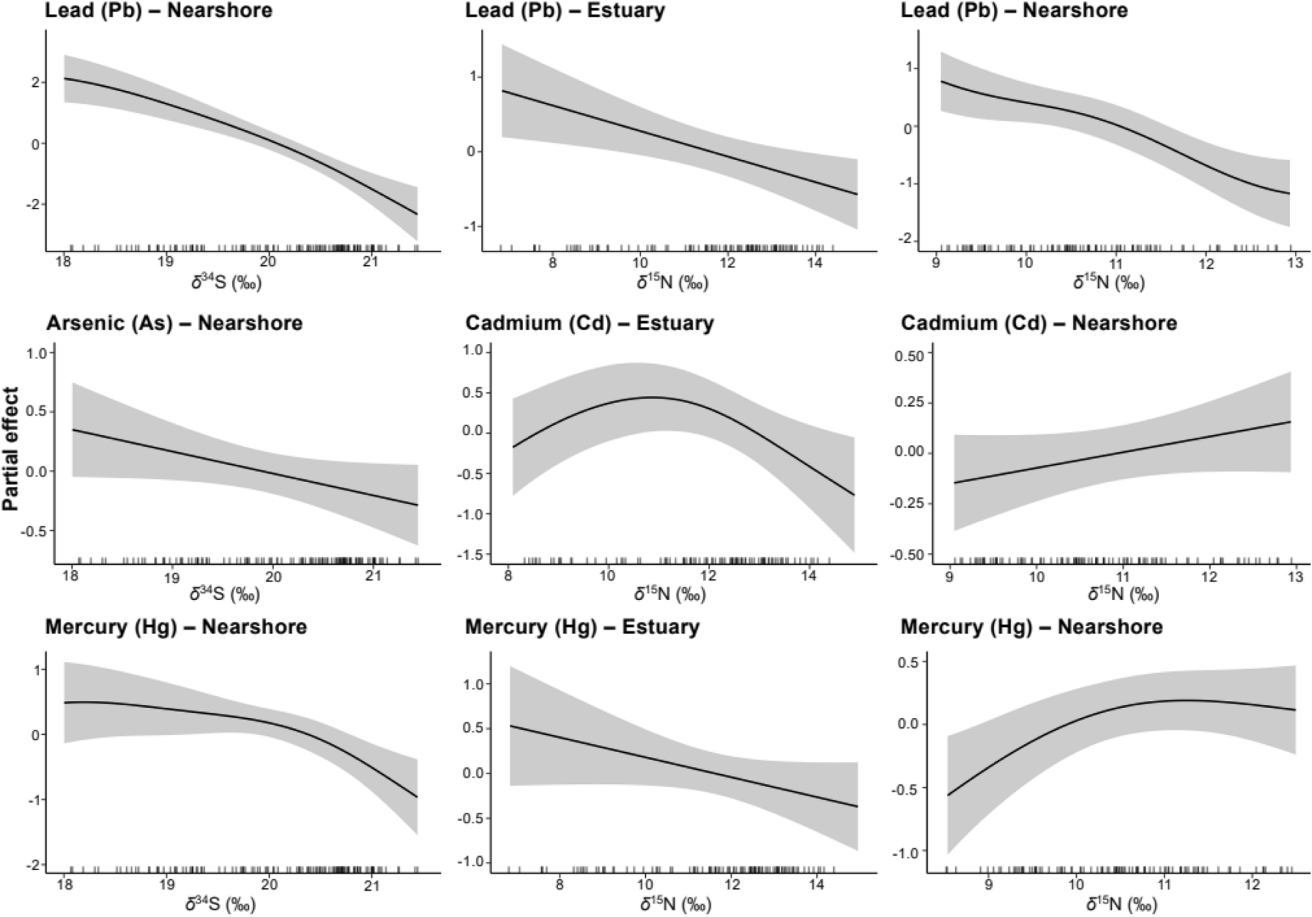
Habitat-specific partial effect smooths of δ^34^S (left graphs) and δ^15^N over nonessential trace elements analyzed in the blood of piscivorous birds sampled in estuarine and nearshore habitats in Brazil, as obtained with generalized additive models.

## 4 Discussion

The gradient from tropical estuaries in southeastern Brazil to the nearshore and offshore southwestern Atlantic Ocean revealed marked spatial variations in trace element concentrations and stable isotope values in the blood of piscivorous waterbirds. The highest concentrations of nonessential elements were detected in estuarine bird samples, suggesting the influence of freshwater discharge of contaminants and dilution toward marine offshore waters. In line with our expectations, sulfur isotope values differed markedly between estuarine and marine habitats and were associated with trace element concentrations in birds, reflecting the spatial variation in contamination across the environmental gradient.

### 4.1 Stable Isotope Variation across Waterbird Assemblages

Sulfur isotopes spanned roughly 20 ‰ (range: 0.38–20.2 ‰) in the estuarine bird assemblage, whereas the variation was much lower in marine habitats, at 5.4 ‰ (range: 16.0–21.4 ‰). Within the estuary, there was a clear differentiation between species feeding in small creeks (green kingfisher; lowest δ^34^S values) from those feeding in the open estuary (Amazon kingfisher, ringed kingfisher *Megaceryle torquata* and black skimmer *Rynchops niger*; intermediate δ^34^S values) and those feeding mainly at sea (e.g., Cabot’s tern *Thalasseus acuflavidus* and common tern *Sterna hirundo*; high marine δ^34^S values). As demonstrated for freshwater fishes, anoxic sediment conditions favor sulfate reduction and lower δ^34^S values in shallow waters due to sulfide input,^37,40^ which may contribute to the lowest δ^34^S values in the green kingfisher. In contrast, δ^34^S values are assumed to be relatively constant in seawater, although lower values can occur in coastal areas influenced by freshwater inputs.^29,52^ In the nearshore habitat, the highest δ^34^S values were recorded in the masked booby *Sula dactylatra* and the red-billed tropicbird *Phaethon aethereus*, both of which using wide-ranging areas on the continental shelf and outward.^16^ The lowest δ^34^S values were recorded in the magnificent frigatebird *Fregata magnificens*, the brown noddy *Anous stolidus* and the brown booby *Sula leucogaster*, with the latter foraging mostly in coastal waters according to tracking data,^16^ whereas frigatebirds are also common along the coast and brown noddies are often recorded between the nearshore site and the mainland (Authors pers. obs.). Therefore, the nearshore seabird assemblage seems to present contrasting δ^34^S values between species foraging near the coast and those foraging farther offshore. In turn, seabirds from the offshore site had high and more homogeneous δ^34^S values, comparable to those observed in nearshore species foraging offshore, which further suggests limited variability in sulfur isotopes in the tropical oceanic waters of the southwestern Atlantic Ocean.

Carbon isotopic values showed less differentiation among waterbird assemblages than sulfur isotopes. The lowest and highest δ^13^C values were measured in the estuary, pointing to high variability in the estuarine bird assemblage (−28.6 to −11.8 ‰). Low δ^13^C values are expected in freshwater ecosystems in comparison to ^13^C-enriched marine phytoplankton.^35^ On the other hand, nearshore waters generally show higher δ^13^C values than the open ocean due to contrasting nutrient loading,^32,33^ and may have contributed to the highest δ^13^C values recorded in some estuarine birds that forage at sea (e.g., black skimmer, common and Cabot’s terns). Nevertheless, with the exception of the offshore Trindade petrel *Pterodroma arminjoniana*, which presented lower δ^13^C values similar to those of estuarine kingfishers, offshore species generally showed δ^13^C values comparable to nearshore species, thereby not evidencing the expected neritic–oceanic pattern in δ^13^C values.^32,33^ These results suggest a limited spatial resolution of δ^13^C in waterbird assemblages across estuarine–oceanic gradients due to the overlap across widely different systems. Therefore, we agree with other studies that recommend the use of sulfur isotopes to represent aquatic bird habitats along coastal gradients due to its linear and predictable variation,^17,36,52^ although a limited contrast between nearshore and offshore marine habitats was also evidenced.

Spatial variation in nitrogen isotope values across waterbird assemblages is likely driven by differences in δ^15^N baselines along the estuary–open ocean gradient rather than by true differences in trophic position. In estuarine species, δ^15^N values increased from birds foraging primarily within the river estuary (lower δ^34^S and δ^13^C values; i.e., kingfishers) to species that also forage at sea (higher δ^34^S and δ^13^C values). In contrast, δ^15^N values decreased markedly from nearshore to offshore seabird assemblages, consistent with the expected decline in δ^15^N baselines toward the open ocean.^32,33^ Among nearshore seabirds, the highest δ^15^N values occurred in species foraging in coastal waters, as indicated by their lower δ^34^S values and available tracking data,^16^ suggesting baseline enrichment near the coast or, alternatively, higher trophic position. Overall, these results highlight that bulk δ^15^N values should be interpreted cautiously when used as indicators of trophic position, particularly when sampling spans multiple species and habitats influenced by contrasting isotopic baselines.^31,33^ Complementary approaches, such as compound-specific isotope analysis of trophic and source amino acids, may help disentangle baseline and trophic effects in waterbirds across broad coastal gradients.^53,54^

### 4.2 Spatial Patterns of Trace Element Contamination in Waterbirds and Relationships with Isotopic Tracers

The concentrations of Cd and Pb were consistently higher in the estuary and showed the strongest negative relationships with δ^34^S, indicating a steep decline in the concentrations associated with the isotopic signature of marine sulfate (Figure 4). Cadmium also demonstrated a nonlinear relationship with δ^13^C, with increasing concentrations associated with the lowest and highest δ^13^C values observed in the estuary. These patterns suggest higher freshwater inputs of Cd and Pb and limited seaward dispersion.^42^ In the Doce River estuary, multiple trace elements (including Cd and Pb) may become trapped in sediments during estuarine mixing due to the formation of colloidal particles with insoluble Fe oxyhydroxides associated with the Fundão Dam tailings.^12,43^ Cadmium is less particle-reactive than Pb and behaves more conservatively in the estuarine mixing zone,^9,13^ making it more bioavailable in the estuary and potentially contributing to the greater spatial contrast in Cd concentrations between estuarine and marine birds compared to Pb, as indicated by the greater explanatory power of the Cd isotope-only model (37.3%), which was more than twice that obtained for Pb (17.9%). In addition to higher bioavailability in the estuary, Cd is depleted in oceanic surface waters due to biological uptake by phytoplankton and subsequent removal with sinking organic matter, potentially contributing to the lower Cd concentrations in offshore seabirds.^9^ Conversely, the long-range atmospheric transport of Pb, enhanced by anthropogenic inputs (e.g., fossil fuel combustion), is the dominant source of to the open ocean, leading to higher Pb concentrations in pelagic food webs.^9^

The results from the GAMs also revealed a negative relationship between δ^34^S and As and Hg, but the results from the PCA suggest that these associations were weaker (Figure 3). In addition, there was no difference between habitats in regard to As, and the concentration of Hg was actually greater in the offshore birds than those in the estuary (medians in Table 1). The lack of a clearer pattern in the difference of As and Hg concentrations among habitats and the weaker relationships with δ^34^S are likely related to factors influencing the concentration and bioavailability of these elements in the ocean. Arsenic occurs predominantly as dissolved arsenate in aquatic systems and is less prone to scavenging in seawater than in estuaries because of the higher pH in seawater.^55^ In addition, marine phytoplankton converts inorganic As into nontoxic organic As compounds (e.g., arsenosugars), which are effectively transferred along marine food chains and excreted by higher consumers.^55^ For Hg, transport to remote oceanic locations through the atmosphere,^9^ together with methylation by marine microorganisms,^36,52^ MeHg biomagnification and longer marine food chains, contribute to higher concentrations in offshore food webs.^19,36^ Accordingly, previous studies assessing δ^34^S and Hg relationships in seabirds have reported positive associations that point to the increase in Hg in oceanic waters, such as in seabirds from the Canadian Pacific coast,^36^ the northern Atlantic Ocean,^15,29^ and the Mediterranean.^23,56^ In Brazilian waters, Benemann et al.^57^ reported higher concentrations of Hg in the feathers of the brown booby in an offshore archipelago than at a nearshore site. Conversely, Góngora et al.^52^ reported a negative relationship between sulfur isotopes and Hg in Arctic seabird prey and attributed this finding partially to geographical variation and proximity to freshwater Hg inputs. The negative relationships of As and Hg concentrations with δ^34^S values in our study are clearly influenced by the highest individual values of these elements recorded in estuarine samples, which are likely explained by continental inputs of these trace elements. Moreover, the negative relationships between δ^15^N values and Hg, as well as with metals that are known to bioreduce (i.e., Cd and Pb), are consistent with different nitrogen isotopic baselines along the estuary–open ocean gradient.

The concentrations of most essential trace elements (Cr, Cu, Mn, and Zn) appeared to be relatively higher in offshore seabirds and positively correlated with δ^34^S (Table 1; Figures 3 and S1). Conversely, the concentrations of Fe were higher in the estuary, which is in line with the Fe mining history of the rivers and the potential influence of the Fundão Dam tailings deposited in the riverbed.^12,42^ Oceanographic processes, such as the upwelling of nutrient-rich deep waters, may increase essential trace element concentrations in offshore seabirds.^58^ In addition, nonessential trace elements may mimic essential elements and compete for binding sites on several proteins that are responsible for the uptake, transport and regulation of essential trace elements in metabolism.^8^ For instance, Cd and Pb are known to disrupt calcium metabolism and displace Zn from metallothioneins, whereas Cr, Cu and Mn can also be affected by similar processes.^8^ Deficiencies in essential elements (e.g., Ca and Zn) may represent an additional concern in areas affected by high concentrations of nonessential trace elements, potentially affecting the immune system, reproduction and development.^7,8^ These results suggest complex interactions between essential and nonessential trace elements across waterbird foraging habitats.

In the habitat-specific models, the nearshore seabird assemblage was the only one that revealed strong negative associations between nonessential trace elements and δ^34^S, consistent with the global patterns. This likely reflects spatial segregation in the foraging areas of seabirds sampled in the nearshore habitat,^16^ with some species feeding mostly in coastal waters (low δ^34^S) and others feeding offshore (high δ^34^S), where a higher contaminant exposure occurs closer to continental sources. Moreover, nearshore birds showed a positive relationship between δ^15^N and Hg, which is consistent with trophic magnification of MeHg,^19^ but potentially also influenced by elevated δ^15^N baselines in coastal waters.^32,33^ A similar relationship between δ^15^N and Cd further supports the influence of baseline structure over δ^15^N-metal relationships in the nearshore assemblage. In contrast, no relationships between δ^34^S and nonessential trace elements were detected in the estuarine dataset, despite the wide range of sulfur isotope values among estuarine species. Nonetheless, δ^15^N was negatively associated with Cd, Hg and Pb in estuarine models, mirroring patterns observed in the global dataset and potentially reflecting an increase in δ^15^N values of the baseline from the inner estuary toward coastal waters, coupled with a decrease in contamination exposure. Negative δ^15^N-Hg relationships reported in other studies have also been partially attributed to the tissue analyzed (e.g., muscle in fishes and cetaceans;^22,59^ ), due to differential MeHg allocation across tissues. Considering that the circulating blood reflects recent exposure and that it may be temporally influenced by individual physiological condition, it is possible that tissues integrating MeHg over longer time windows could show stronger Hg biomagnification patterns, such as feather, liver and kidney samples.

In addition to the spatial structure evidenced in our results, trace element exposure in estuarine and nearshore systems is known to vary temporally, as demonstrated for several organisms in the study area, including nearshore seabirds.^26,45,60^ In the Doce River estuary, hydrological variability can modulate trace element bioavailability through processes such as sediment resuspension and redox-driven remobilization of metals associated with Fe oxyhydroxides, contributing to short-term variability in exposure.^11,12,41^ The PCA results showed that Fe in bird blood covaried with Cd and Pb and was negatively associated with δ^34^S values, highlighting the coupled variability of these metals in relation to freshwater inputs. The temporal heterogeneity of the data set used in this study further integrates periods of contrasting hydrological and environmental conditions, which likely contributes to the wide range and the highest concentrations of several trace elements observed in estuarine birds (Table 1 and Figure 3). Accordingly, the first PCA component captured substantial variation in trace element data that was weakly associated with spatial structure, which is potentially associated with temporal variation in contaminant exposure, as well as individual-level factors not accounted for in this study (e.g., breeding status, age). Moreover, the GAMs indicated that species-specific temporal structure accounted for a substantial proportion of variation in nonessential trace element data. In this context, using multiple species within each habitat therefore integrates this background variability across species and sampling periods rather than emphasizing species-specific or event-driven responses. The consistent habitat-level patterns observed across elements indicate that combining species to represent distinct waterbird assemblages enables assessment of broad-scale variations in trace element contamination.

Toxicological risks can be estimated for waterbirds in this study by comparing the concentrations of nonessential trace elements with the toxicity benchmarks proposed for bird blood in previous studies. With respect to Cd, a blood concentration of 0.33 mg/L was reported in mallard ducks (*Anas platyrhynchos*) fed with food containing 200 ppm of Cd for 90 days, causing several types of microscopic damage to the testis and kidneys.^61,62^ Values above this benchmark for Cd were recorded in 25.5% of the estuarine samples and in two samples (1.6%) from the nearshore site (Table 2). Notably, Cd concentrations in estuarine birds were up to 10 times this reference value, raising concern because of the high toxicity of this element. In terms of Pb contamination, poisoning may be evidenced by tissue lesions, anemia, muscular incoordination and diarrhea.^63^ Published benchmarks of Pb poisoning indicate subclinical poisoning in 7.2–15.1% of waterbird samples across the habitats, whereas 7.5% of the samples from the estuary were above the levels that suggest clinical poisoning, and 6.6% indicated severe clinical poisoning^63^ (Table 2). Regarding Hg, when the concentrations in our study were compared with the risk categories proposed by Ackerman et al.,^64^ a moderate toxicological risk was attributed to 3.8% of the individuals from the estuary and 1.2% from the offshore habitat; whereas 5.7% of the estuarine birds were at levels suggesting a severe toxicological risk from Hg exposure (>4 mg/L), with concentrations up to two times this reference value. Finally, for As, some studies have used the reference concentration of 0.02 mg/L reported by Burger and Gochfeld^65^ in the blood of gulls sampled at an unpolluted site as a threshold of natural exposure,^6,66^ whereas the same study reported up to 0.5 mg/L As in the blood of gulls at an urban site. Considering the As benchmark of 0.5 mg/L in urban gulls, 40.6% of the individuals were above that in the estuary, 28.8% in the nearshore and 84.8% in the offshore habitat (Table 2). Although a relatively large proportion of samples from the offshore site were above the reference value, it may not represent a real toxicological risk for seabirds because As in the ocean is largely in the form of nontoxic organoarsenic compounds,^55^ as mentioned previously. Furthermore, the highest As concentrations were found in estuarine (up to 14.9 mg/L) and nearshore birds (up to 3.9 mg/L), with a lower range in offshore birds (up to 1 mg/L). Toxicological benchmarks used here for comparison should be interpreted cautiously, as experimental studies linking adverse effects to blood concentrations are limited for birds—particularly seabirds—and such relationships may vary substantially across taxa. Nevertheless, the quantification of all nonessential trace elements above established benchmarks for toxicity or natural exposure suggests potentially hazardous contamination in piscivorous waterbirds in Brazil, particularly in estuaries.

**Table 2:** Percentage Waterbird Samples from Habitat That Presented Blood Concentrations of Nonessential Trace Elements (As, Cd, Hg and Pb) above a Benchmark for Impact/Toxicity, Established for Each Element in Previous Studies

Element	Benchmark or risk category	Concentration (mg/L ww)	Reference	Estuary	Nearshore	Offshore
Arsenic	Concentration in urban gulls from New York, USA	0.5	Burger and Gochfeld^65^	40.6	28.8	45.9
Cadmium	Tissue damage in mallard ducks fed experimentally	0.33	White and Finley;^62^ White et al.^61^	25.5	1.6	0
Mercury	Low risk	0.20–1.0	Ackerman et al.^64^	12.3	14.4	24.7
	Moderate risk	1.0–3.0		3.8	0	1.2
	High risk	3.0–4.0		0	0	0
	Severe risk	>4.0		5.7	0	0
Lead	Subclinical poisoning	0.2–0.5	Franson and Pain^63^	15.1	7.2	2.4
	Clinical poisoning	0.5–1.0		7.5	2.4	0
	Severe poisoning	>1.0		6.6	0	0

Through the analysis of a suite of essential and nonessential trace elements and stable isotopes in the blood of waterbirds from estuaries to the open ocean, we were able to assess broad-scale environmental patterns of aquatic pollution affecting wildlife in the tropical southwestern Atlantic Ocean. Our results reveal higher concentrations of all nonessential trace elements in estuaries and a decrease in concentrations in marine offshore waters, as evidenced by the relationships with sulfur isotopes. High concentrations of multiple trace elements simultaneously, exceeding available element-specific benchmarks, raise concern due to potential additive or synergistic effects among elements, thereby increasing toxicological risks, especially for estuarine birds. These results reinforce the inference that birds foraging in areas contaminated by the Fundão Dam tailings may be in an ecological trap, as they continue to forage in areas degraded with high concentrations of several toxic trace elements.^12,14,44–47^ Chronic contamination due to the release from resuspension of contaminated sediments highlights the necessity of continued monitoring of contaminants in aquatic wildlife inhabiting dynamic coastal environments, as well as assessing effects on bird populations and other long-lived, wide-ranging species. Moreover, these findings underscore the need for broad-scale sampling of aquatic predators in contaminated areas and the use of effective habitat tracers to determine the spatial extent of environmental pollution in aquatic species and ecosystems. In addition to spatial variation in contaminant exposure, temporal and individual-level variability could also be explored in future studies.

## Supplementary Material



## Notes

Additional sampling details; materials and methods; tables and figures with trace element and stable isotope data; model results (PDF)
