# Correction to “Sulfur Isotopes Reveal Spatial Variation in Waterbird Trace Element Contamination from Tropical Estuaries to the Open Ocean”

**DOI:** 10.1021/acs.est.6c10313

**Published:** 2026-08-03

**Authors:** Bruno A. Linhares, Paco Bustamante, Guilherme T. Nunes, Patrícia G. Costa, Leandro Bugoni, Yuri D. Zebral, Iole B. M. Orselli, Camila M. G. Martins, Adalto Bianchini

A corrected version of Table S1 appears in the revised Supporting Information presented here. The headers now match the values presented. This error was exclusive of Table S1 and had no influence in the data, analyses, results, and interpretations in the paper.

## Supplementary Material



## Notes

Additional sampling details, materials and methods, tables and figures with trace element and stable isotope data, and model results (PDF)

