# Supplementary material for: Correction to “Sulfur Isotopes Reveal Spatial Variation in Waterbird Trace Element Contamination from Tropical Estuaries to the Open Ocean”
Source: Environ Sci Technol. 2026 Aug 3;60(32):23006. doi: 10.1021/acs.est.6c10313 (PMC13492267; doi:10.1021/acs.est.6c10313)
Supplement: Supplementary file 1 [file es6c10313_si_001.pdf]

SUPPLEMENTARY INFORMATION

Sulfur Isotopes Reveal Spatial Variation in Waterbird Trace Element Contamination from Tropical Estuaries to the Open Ocean

Bruno A. Linhares[1,2], Paco Bustamante[2,3], Guilherme T. Nunes[4], Patrícia G. Costa[5], Leandro Bugoni[1,6], Yuri D. Zebral[7], Iole B. M. Orselli[5], Camila M. G. Martins[7], Adalto Bianchini[1,5,7]*

[1] Programa de Pós-Graduação em Oceanografia Biológica, Instituto de Oceanografia, Universidade Federal do Rio Grande – FURG, 96203-900, Rio Grande, RS, Brazil

[2] Littoral Environnement et Sociétés (LIENSs), UMR 7266 CNRS - La Rochelle Université, 17000 La Rochelle, France

[3] Institut Universitaire de France (IUF), 1 rue Descartes 75005 Paris, France

[4] Centro de Estudos Costeiros, Limnológicos e Marinhos, Universidade Federal do Rio Grande do Sul – UFRGS, 95625-000, Imbé, RS, Brazil

[5] Laboratório de Determinações 2, Instituto de Ciências Biológicas, Universidade Federal do Rio Grande – FURG, 96203-900, Rio Grande, RS, Brazil

[6] Laboratório de Aves Aquáticas e Tartarugas Marinhas, Instituto de Ciências Biológicas, Universidade Federal do Rio Grande – FURG, 96203-900, Rio Grande, RS, Brazil

[7] Programa de Pós-Graduação em Ciências Fisiológicas, Instituto de Ciências Biológicas, Universidade Federal do Rio Grande – FURG, 96203-900, Rio Grande, RS, Brazil

[*] *Corresponding author: brunolinhares.bio@gmail.com*

Sampling and species detail

Blood samples were collected from 15 species of waterbirds across estuarine, nearshore and offshore habitats in southeastern Brazil and the southwestern Atlantic Ocean (Table 1 and Fig. 1 in the main text for specific locations). Across estuaries, kingfishers (Coraciiformes; Alcedinidae), skimmers (Charadriiformes; Rynchopidae) and terns (Charadriiformes; Sternidae) were captured using mistnets placed along estuaries. Amazon (*Chloroceryle amazona*; *n* = 29), green (*C. americana*; *n* = 16) and ringed (*Megaceryle torquata*; *n* = 9) kingfishers are three common resident freshwater species along rivers in Brazil and are considered estuarine/freshwater foragers based on known ecology. Black skimmers (*Rynchops niger*; *n* = 16) forage mainly along estuaries but may also forage in shallow coastal waters. While Cabot's terns (*Thalasseus acuflavidus*; *n* = 17) breed on islands along the coast in southeastern Brazil, common terns (*Sterna hirundo*; *n* = 19) are transequatorial migrants that breed near the Arctic. Both terns use estuaries in southeastern Brazil extensively during the non-breeding period, and are assumed to represent a mix of estuarine and marine signatures. Samples in the estuarine habitat were collected during winter and summer seasons between 2018 and 2024 as part of the Aquatic Biodiversity Monitoring Program of the Doce River and Adjacent Coastal and Marine Regions (PMBA) created to monitor the impacts of the Fundão Dam failure.

In the nearshore habitat, the brown noddy (Charadriiformes; Sternidae; *Anous stolidus*; *n* = 24), red-billed tropicbird (Phaethontiformes; Phaethontidae; *Phaethon aethereus*; *n* = 23), brown booby (Suliformes; Sulidae; *Sula leucogaster*; *n* = 27), masked booby (*S. dactylatra*; *n* = 23, respectively) and the magnificent frigatebird (Suliformes; Fregatidae; *Fregata magnificens*; *n* = 28) were captured by hand, with handle nets or with a nylon noose attached to a fishing pole. Niche partitioning is expected among these seabird species primarily due to differences in diet and foraging areas, such as species feeding in coastal waters (brown booby) and species feeding more in oceanic waters (masked booby, tropicbird) (see [1]). Samples of brown and masked boobies and tropicbirds were collected in summer and winter seasons as part of the PMBA between 2022 and 2024. Samples from the brown noddy and frigatebirds were collected in 2011 and 2024.

In the offshore habitat, at approximately 1,200 km from the Brazilian coast, blood samples were collected using the same methods reported for nearshore seabirds. Seabirds sampled included the white tern (*Gygis alba*; *n* = 16), sooty tern (*Onychoprion fuscatus*; *n* = 19), brown noddy (*n* = 11), masked booby (*n* = 19) and the Trindade petrel (Procellariiformes; Procellariidae; *Pterodroma arminjoniana*; n = 20). Segregation in isotopic niches was demonstrated for these seabirds in previous studies [2]. The Trindade petrel uses a wide oceanic region and migrates to the northern hemisphere during the non-

breeding season [3]. Samples of the offshore seabirds were collected in 2007 (masked booby, terns), 2017 (sooty tern) and 2022 (except sooty terns).

All species included in the study are piscivorous (fish-eating), although some seabirds may also consume squids [1,3]. Multiple species were selected to represent piscivorous bird assemblages in each habitat, covering a range of foraging habitats, feeding strategies and taxonomic groups. Seabird species utilize nearshore and offshore sampling localities as breeding sites; kingfishers and the black skimmer potentially breed along the estuaries. Adult or subadult individuals were randomly sampled, and individual-level information such as breeding status, age, molt and sex were not consistently obtained, and therefore were not considered in analysis. Captured birds were handled following standard protocols to minimize stress and handling time, and released near the site of capture; blood samples collected with syringe and needle were stored frozen until analysis in the Laboratório de Determinações 2, at the Universidade Federal do Rio Grande (FURG). Sampling was allowed by environmental agencies and universities' ethics committees. Additional collection details are reported elsewhere [1,4–7].

REFERENCES

(1) Linhares, B. A.; Nunes, G. T.; Bianchini, A.; Bertolini, L.; Vilela, F.; Efe, M. A.; Rodrigues, F. L.; Lanco, S.; Zebral, Y. D.; Costa, P. G.; Bugoni, L. Resource Partitioning Influences Levels of Toxic Trace Elements in Sympatric Tropical Seabirds. *Sci. Total Environ.* **2024**, *949*, 175102. https://doi.org/10.1016/j.scitotenv.2024.175102.

(2) Mancini, P. L.; Hobson, K. A.; Bugoni, L. Role of Body Size in Shaping the Trophic Structure of Tropical Seabird Communities. *Mar. Ecol. Prog. Ser.* **2014**, *497*, 243–257. https://doi.org/10.3354/meps10589.

(3) Leal, G. R.; Furness, R. W.; McGill, R. A. R.; Santos, R. A.; Bugoni, L. Feeding and Foraging Ecology of Trindade Petrels *Pterodroma arminjoniana* during the Breeding Period in the South Atlantic Ocean. *Mar. Biol.* **2017**, *164* (11), 211. https://doi.org/10.1007/s00227-017-3240-8.

(4) Bauer, A. D. B.; Linhares, B. A.; Nunes, G. T.; Costa, P. G.; Zebral, Y. D.; Bianchini, A.; Bugoni, L. Temporal Changes in Metal and Arsenic Concentrations in Blood and Feathers of Tropical Seabirds after One of the Largest Environmental Disasters Associated with Mining. *Environ. Res.* **2024**, *248*, 118240. https://doi.org/10.1016/j.envres.2024.118240.

(5) Nunes, G. T.; Efe, M. A.; Barreto, C. T.; Gaiotto, J. V.; Silva, A. B.; Vilela, F.; Roy, A.; Bertrand, S.; Costa, P. G.; Bianchini, A.; Bugoni, L. Ecological Trap for Seabirds Due to the Contamination Caused by the Fundão Dam Collapse, Brazil. *Sci. Total Environ.* **2022**, *807*, 151486. https://doi.org/10.1016/j.scitotenv.2021.151486.

(6) Linhares, B. D. A.; Costa, P. G.; Bugoni, L.; Nunes, G. T.; Bianchini, A. Concentrations of Organic Pollutants in Seabirds from the Tropical Southwestern Atlantic Ocean Are Explained by Differences in Foraging Ecology. *Environ. Pollut.* **2025**, *371*, 125928. https://doi.org/10.1016/j.envpol.2025.125928.

(7) Zebral, Y. D.; Costa, P. G.; De Souza, M. M.; Bianchini, A. Avian Blood and Feathers as Biological Tools to Track Impacts from Trace-Metals: Bioaccumulation Data from the Biggest Environmental Disaster in Brazilian History. *Sci. Total Environ.* **2022**, *807*, 151077. https://doi.org/10.1016/j.scitotenv.2021.151077.

Table S1. Mean ± standard deviation and range (minimum, maximum) of the concentrations (mg/L ww) of trace elements and values of stable isotopes (‰) of carbon ($\delta^{13}C$), nitrogen ($\delta^{15}N$) and sulfur ($\delta^{34}S$) in the blood of estuarine and marine birds sampled in estuaries in southeastern Brazil and islands in the southwestern Atlantic Ocean.

	n	years	$\delta^{13}C$	$\delta^{15}N$	$\delta^{34}S$	As	Cd	Hg	Pb	Cr	Cu	Fe	Mn	Zn
**Estuary**	106	2018-2024	-20.43 ± 3.31	11.57 ± 2.25	13.34 ± 4.85	1.05 ± 2.07	0.31 ± 0.51	0.62 ± 1.85	0.24 ± 0.38	1.84 ± 3.38	1.57 ± 4.61	160.26 ± 603.67	5.69 ± 16	10.16 ± 27.1
			(-28.59, -11.76)	(6.27, 18.29)	(0.38, 20.2)	(0.002, 14.91)	(0.004, 3.31)	(0.003, 8.48)	(0.005, 1.84)	(0.002, 21.12)	(0.004, 33.74)	(0.13, 5468.91)	(0.03, 146.19)	(0.05, 186.43)
Chloroceryle americana (Green kingfisher)	16	2018-2024	-25.27 ± 1.75	9.14 ± 2.26	7.35 ± 5.05	1.49 ± 2.09	0.51 ± 0.94	0.33 ± 0.76	0.24 ± 0.36	2.26 ± 4.34	1.40 ± 2.3	258.37 ± 572.43	7.63 ± 13.79	21.15 ± 46.42
			(-28.59, -21.52)	(6.27, 15.43)	(0.38, 16.12)	(0.02, 6.54)	(0.01, 3.31)	(0.004, 2.93)	(0.005, 1.42)	(0.06, 17.94)	(0.04, 8.18)	(3.05, 2091.67)	(0.06, 53.91)	(0.55, 186.43)
Chloroceryle amazona (Amazon kingfisher)	29	2019-2024	-20.84 ± 3.34	11.41 ± 2.05	11.73 ± 1.98	0.95 ± 1.21	0.32 ± 0.38	0.45 ± 1.04	0.28 ± 0.39	1.65 ± 2.03	0.76 ± 1.07	265.18 ± 1016.69	3.86 ± 5.53	6.57 ± 8.88
			(-28.01, -11.76)	(7.06, 15.09)	(8.12, 15)	(0.008, 4.54)	(0.008, 1.69)	(0.006, 5.17)	(0.005, 1.38)	(0.005, 8.12)	(0.004, 4.98)	(0.19, 5468.91)	(0.11, 20.95)	(0.18, 47.59)
Megaceryle torquata (Ringed kingfisher)	9	2018-2024	-20.84 ± 2.7	10.52 ± 2.3	10.93 ± 3.59	0.95 ± 1.83	0.39 ± 0.42	0.36 ± 0.84	0.18 ± 0.22	2.73 ± 2.07	1.54 ± 2.42	392.48 ± 532.63	26.22 ± 53.25	20.55 ± 40.59
			(-23.49, -16.02)	(6.81, 13.36)	(2.01, 13.32)	(0.002, 5.79)	(0.02, 1.09)	(0.008, 2.59)	(0.005, 0.73)	(0.012, 5.17)	(0.12, 7.04)	(0.90, 1540)	(0.23, 146.19)	(1.17, 127.94)
Rynchops niger (Black skimmer)	16	2019-2024	-17.82 ± 3.05	13.2 ± 2.56	12.49 ± 2.73	0.14 ± 0.17	0.04 ± 0.05	0.06 ± 0.11	0.13 ± 0.21	1.21 ± 3.23	0.51 ± 0.59	39.04 ± 114.18	3.48 ± 8.79	11.67 ± 39.87
			(-23.26, -13.83)	(8.33, 18.29)	(7.54, 16.74)	(0.01, 0.68)	(0.008, 0.15)	(0.003, 0.44)	(0.005, 0.74)	(0.002, 13.05)	(0.007, 1.58)	(0.13, 461.52)	(0.03, 36.08)	(0.05, 161.04)
Sterna hirundo (Common tern)	19	2022-2024	-19.36 ± 1	11.96 ± 0.95	18.21 ± 2.04	1.76 ± 3.57	0.42 ± 0.57	1.36 ± 3.07	0.42 ± 0.58	2.19 ± 4.82	4.69 ± 10.09	26.23 ± 46.79	3.98 ± 4.9	5.8 ± 5.88
			(-21.62, -18.23)	(9.05, 13.08)	(11.07, 19.52)	(0.15, 14.91)	(0.005, 2.08)	(0.008, 8.43)	(0.01, 1.84)	(0.024, 21.12)	(0.07, 33.74)	(0.67, 195.79)	(0.20, 16.86)	(0.50, 19.73)
Thalasseus acuflavidus (Cabot's tern)	17	2019-2024	-18.65 ± 0.76	12.63 ± 0.89	18.36 ± 2.37	0.94 ± 1.92	0.18 ± 0.25	1 ± 2.74	0.13 ± 0.26	1.56 ± 3.22	0.70 ± 0.98	29.90 ± 69.81	2.35 ± 2.3	3.87 ± 5.96
			(-21.00, -17.52)	(10.28, 14.39)	(12.34, 20.2)	(0.06, 7.29)	(0.004, 0.8)	(0.004, 8.48)	(0.005, 1.1)	(0.007, 13.55)	(0.06, 3.67)	(0.13, 292.1)	(0.15, 8.81)	(0.07, 22.35)
**Abrolhos (nearshore)**	125	2011-2024	-17.78 ± 0.9	10.93 ± 1.6	19.89 ± 1.09	0.42 ± 0.44	0.03 ± 0.06	0.11 ± 0.1	0.08 ± 0.15	3.4 ± 4.41	4.9 ± 9.61	7.57 ± 8.65	6.37 ± 11.19	13.82 ± 23.57
			(-19.91, -15.68)	(7.43, 14.7)	(15.95, 21.44)	(0.009, 3.96)	(0.005, 0.37)	(0.004, 0.42)	(0.002, 0.88)	(0.008, 14.61)	(0.061, 95.54)	(0.117, 48.18)	(0.052, 79.82)	(0.028, 98.46)
Anous stolidus (Brown noddy)	24	2011, 2024	-18.82 ± 0.49	10.91 ± 0.4	19.51 ± 0.35	0.45 ± 0.21	0.02 ± 0.02	0.15 ± 0.12	0.07 ± 0.1	7.16 ± 5.07	6.19 ± 6.42	6.33 ± 3.66	9.47 ± 12.56	7.49 ± 7.24
			(-19.91, -18.24)	(10.35, 11.74)	(18.91, 20.3)	(0.04, 0.81)	(0.005, 0.06)	(0.004, 0.39)	(0.02, 0.34)	(0.008, 14.61)	(0.06, 28.44)	(0.14, 13.02)	(0.10, 40.9)	(0.20, 21.61)
Phaethon aethereus (Red-billed tropicbird)	23	2022-2024	-17.94 ± 0.44	9.48 ± 1.1	20.89 ± 0.27	0.3 ± 0.25	0.02 ± 0.01	0.09 ± 0.12	0.06 ± 0.06	0.93 ± 1.38	2.89 ± 4.72	9.18 ± 10.45	5.55 ± 16.84	3.62 ± 5.82
			(-18.85, -17.11)	(7.43, 11.75)	(20.37, 21.44)	(0.016, 0.82)	(0.007, 0.05)	(0.004, 0.4)	(0.002, 0.18)	(0.06, 5.71)	(0.09, 21.18)	(0.12, 30.56)	(0.08, 79.82)	(0.03, 21.54)
Sula leucogaster (Brown booby)	27	2022-2024	-17.96 ± 0.56	11.22 ± 1.06	20.18 ± 0.55	0.33 ± 0.27	0.04 ± 0.07	0.13 ± 0.1	0.1 ± 0.17	0.99 ± 1.36	3.98 ± 5.08	10.04 ± 11.55	5.13 ± 8.33	4.86 ± 7.4
			(-19.59, -17.12)	(9.87, 13.52)	(18.52, 20.83)	(0.009, 1.34)	(0.005, 0.34)	(0.005, 0.42)	(0.002, 0.88)	(0.08, 4.8)	(0.10, 14.8)	(0.12, 48.18)	(0.08, 37.16)	(0.22, 30.77)
Sula dactylatra (Masked booby)	23	2022-2024	-17.33 ± 0.52	9.66 ± 0.78	20.74 ± 0.26	0.24 ± 0.2	0.03 ± 0.02	0.06 ± 0.05	0.03 ± 0.05	1.92 ± 3.14	6.62 ± 20.49	3.7 ± 4.25	4.34 ± 7.89	16.82 ± 29.71
			(-18.56, -16.42)	(8.09, 11.86)	(20.27, 21.26)	(0.013, 0.72)	(0.005, 0.06)	(0.004, 0.18)	(0.002, 0.23)	(0.008, 8.89)	(0.31, 95.54)	(0.12, 16.9)	(0.13, 26.49)	(0.03, 85.98)
Fregata magnificens (Magnificent frigatebird)	28	2011, 2024	-16.92 ± 0.93	12.89 ± 1.35	18.42 ± 1.02	0.70 ± 0.76	0.05 ± 0.09	0.12 ± 0.06	0.13 ± 0.22	5.72 ± 5	4.98 ± 4.23	8.09 ± 8.79	7.14 ± 9	33.78 ± 32.89
			(-19.71, -15.68)	(9.12, 14.7)	(15.95, 20.7)	(0.021, 3.96)	(0.005, 0.37)	(0.014, 0.25)	(0.005, 0.88)	(0.01, 12.18)	(0.06, 14.69)	(0.41, 33.23)	(0.05, 31.83)	(0.05, 98.46)
**Trindade (offshore)**	85	2007-2022	-17.82 ± 0.86	9.2 ± 1.37	20.72 ± 0.42	0.43 ± 0.31	0.01 ± 0.01	0.17 ± 0.31	0.04 ± 0.05	6.9 ± 4.65	3.05 ± 5.9	7.91 ± 7.34	11.24 ± 10.28	12.77 ± 10.95

	n	years	$\delta^{13}C$	$\delta^{15}N$	$\delta^{34}S$	As	Cd	Hg	Pb	Cr	Cu	Fe	Mn	Zn
			(-19.42, -16.47)	(7.00, 11.92)	(19.58, 21.33)	(0.002, 1)	(0.004, 0.04)	(0.004, 2.73)	(0.006, 0.29)	(0.008, 14.1)	(0.05, 34.16)	(0.39, 42.99)	(0.08, 42.78)	(0.11, 56.15)
Anous stolidus (Brown noddy)	11	2007, 2022	-17.51 ± 0.19	8.05 ± 0.31	20.07 ± 0.22	0.51 ± 0.27	0.01 ± 0.01	0.21 ± 0.04	0.03 ± 0.01	11.17 ± 1.34	5.65 ± 6.97	15.58 ± 9.74	14.49 ± 6.86	17.02 ± 4.82
			(-17.99, -17.24)	(7.63, 8.44)	(19.88, 20.58)	(0.002, 0.8)	(0.004, 0.04)	(0.16, 0.31)	(0.02, 0.04)	(9.42, 14.1)	(0.17, 25.22)	(8.13, 42.99)	(3.33, 23.85)	(8.09, 24.58)
Onychoprion fuscatus (Sooty tern)	19	2007, 2017	-17.84 ± 0.56	8.22 ± 0.79	20.93 ± 0.24	0.58 ± 0.24	0.01 ± 0.01	0.35 ± 0.61	0.02 ± 0.01	9.71 ± 2.41	1.62 ± 2.87	11.1 ± 4.21	13.35 ± 9.26	14.04 ± 5.98
			(-18.45, -16.59)	(7, 9.6)	(20.27, 21.27)	(0.12, 1)	(0.005, 0.04)	(0.04, 2.73)	(0.01, 0.04)	(2.61, 13.55)	(0.06, 11.83)	(1.96, 20.3)	(1.28, 31.7)	(3.11, 25.36)
Gygis alba (White tern)	16	2007, 2022	-17.23 ± 0.46	8.63 ± 0.94	20.73 ± 0.15	0.62 ± 0.19	0.01 ± 0.01	0.17 ± 0.04	0.03 ± 0.01	9.53 ± 1.98	7.13 ± 9.42	8.47 ± 8.76	11.33 ± 7.64	15.88 ± 8.8
			(-18.00, -16.47)	(7.78, 11.7)	(20.50, 21.03)	(0.27, 0.9)	(0.004, 0.03)	(0.09, 0.27)	(0.01, 0.05)	(4.75, 12.77)	(0.31, 34.16)	(2.97, 39.81)	(0.37, 22.58)	(2.45, 31.14)
Sula dactylatra (Masked booby)	19	2007, 2022	-17.12 ± 0.3	9.17 ± 0.66	20.40 ± 0.3	0.5 ± 0.26	0.01 ± 0.01	0.14 ± 0.08	0.02 ± 0.01	6.58 ± 3.65	1.22 ± 2.52	6.68 ± 4.47	18.67 ± 11.41	18.42 ± 15.69
			(-17.66, -16.6)	(7.86, 10.12)	(19.58, 20.75)	(0.1, 0.9)	(0.005, 0.03)	(0.04, 0.35)	(0.006, 0.03)	(2.29, 13.01)	(0.10, 8.36)	(1.43, 15.15)	(2.86, 42.78)	(2.77, 56.15)
Pterodroma arminjoniana (Trindade petrel)	20	2022	-19.12 ± 0.2	11.23 ± 0.42	21.14 ± 0.12	0.04 ± 0.01	0.01 ± 0	0.02 ± 0.03	0.1 ± 0.07	0.07 ± 0.06	0.14 ± 0.17	1.37 ± 1.06	0.31 ± 0.2	1.38 ± 0.83
			(-19.42, -18.6)	(10.34, 11.92)	(20.90, 21.33)	(0.02, 0.07)	(0.007, 0.02)	(0.004, 0.08)	(0.03, 0.29)	(0.01, 0.25)	(0.05, 0.52)	(0.39, 4.34)	(0.08, 0.83)	(0.11, 3.44)

Table S2. Summary results from Generalized Additive Models with log-transformed concentrations of trace elements as response variables, considering bird blood samples collected in estuaries, nearshore (Abrolhos Archipelago) and offshore (Trindade Island) marine environments. Significant p-values are presented in bold.

**Generalized Additive Model results**				
**Lead (Pb)**	Effect. Df	Res. Df	F	**p-value**
$\delta^{13}C$	0	9	0	0.597
$\delta^{15}N$	0.93	9	1.383	**<0.001**
$\delta^{34}S$	3.79	9	2.76	**<0.001**
Year/season:species	25.1	65	2.303	**<0.001**
	R2 = 0.396	Dev. Explained = 45.4 %		
**Cadmium (Cd)**	Effect. Df	Res. Df	F	**p-value**
$\delta^{13}C$	2.449	9	1.78	**<0.001**
$\delta^{15}N$	2.194	9	1.157	**0.001**
$\delta^{34}S$	1.835	9	0.767	**0.005**
Year/season:species	18.378	62	1.881	**<0.001**
	R2 = 0.516	Dev. Explained = 55.4 %		
**Arsenic (As)**	Effect. Df	Res. Df	F	**p-value**
$\delta^{13}C$	0.00257	9	0	0.312
$\delta^{15}N$	0.97566	9	0.2	0.116
$\delta^{34}S$	4.29734	9	2.213	**<0.001**
Year/season:species	22.88348	65	1.986	**<0.001**
	R2 = 0.387	Dev. Explained = 44.2 %		
**Mercury (Hg)**	Effect. Df	Res. Df	F	**p-value**
$\delta^{13}C$	0.4195	9	0.08	0.166
$\delta^{15}N$	0.7484	9	0.331	0.032
$\delta^{34}S$	3.77	9	2.393	**<0.001**
Year/season:species	31.1886	65	4.44	**<0.001**
	R2 = 0.533	Dev. Explained = 58.7 %		

Table S3 Summary results from Generalized Additive Models with log-transformed concentrations of trace elements as response variables, considering bird blood samples collected in estuarine habitats in southeast Brazil. Significant p-values are presented in bold.

**Generalized Additive Models – Estuary**				
**Lead (Pb)**	Effect. Df	Res. Df	F	**p-value**
$\delta^{13}C$	0	9	0	0.887
$\delta^{15}N$	0.89	9	0.898	**0.003**
$\delta^{34}S$	0	9	0	0.919
Year/season:species	8.25	37	0.919	**<0.001**
	$R^2 = 0.315$	Dev. Explained = 37.6 %		
**Cadmium (Cd)**	Effect. Df	Res. Df	F	**p-value**
$\delta^{13}C$	0.25	9	0	0.387
$\delta^{15}N$	2.26	9	1.095	**0.003**
$\delta^{34}S$	0	9	0	0.957
Year/season:species	7.65	37	0.406	**<0.001**
	$R^2 = 0.236$	Dev. Explained = 31 %		
**Arsenic (As)**	Effect. Df	Res. Df	F	**p-value**
$\delta^{13}C$	0	9	0.122	0.136
$\delta^{15}N$	0.99	9	0.394	0.068
$\delta^{34}S$	5.19	9	0	0.635
Year/season:species	23.07	37	1.245	**<0.001**
	$R^2 = 0.387$	Dev. Explained = 48.2 %		
**Mercury (Hg)**	Effect. Df	Res. Df	F	**p-value**
$\delta^{13}C$	0	9	0	0.470
$\delta^{15}N$	0.73	9	0.305	**0.041**
$\delta^{34}S$	0	9	0	**0.335**
Year/season:species	23.33	37	4.047	**<0.001**
	$R^2 = 0.615$	Dev. Explained = 70.6 %		

Table S4 Summary results from Generalized Additive Models with log-transformed concentrations of trace elements as response variables, considering bird blood samples collected in the marine nearshore habitat (Abrolhos Archipelago). Significant p-values are presented in bold.

**Generalized Additive Models – Nearshore**				
**Lead (Pb)**	Effect. Df	Res. Df	F	**p-value**
$\delta^{13}C$	0.6371	4	0.439	0.066
$\delta^{15}N$	2.8981	4	5.58	**<0.001**
$\delta^{34}S$	2.7002	4	10.527	**<0.001**
Year/season:species	6.2134	17	3.299	**<0.001**
	R2 = 0.482	Dev. Explained = 53.4 %		
**Cadmium (Cd)**	Effect. Df	Res. Df	F	**p-value**
$\delta^{13}C$	0.6157	2	0.801	0.099
$\delta^{15}N$	0.6787	2	1.056	**0.047**
$\delta^{34}S$	0.9373	4	0.495	0.084
Year/season:species	3.0826	17	3.127	**<0.001**
	R2 = 0.311	Dev. Explained = 34.1 %		
**Arsenic (As)**	Effect. Df	Res. Df	F	**p-value**
$\delta^{13}C$	0.96	4	0.41	0.107
$\delta^{15}N$	0	4	0	0.674
$\delta^{34}S$	0.76	4	0.806	**0.016**
Year/season:species	4.95	17	0.846	**0.004**
	R2 = 0.208	Dev. Explained = 25 %		
**Mercury (Hg)**	Effect. Df	Res. Df	F	**p-value**
$\delta^{13}C$	0	4	0	0.631
$\delta^{15}N$	2.25	4	1.997	**0.011**
$\delta^{34}S$	2.22	4	2.827	**0.002**
Year/season:species	7.77	17	5.816	**<0.001**
	R2 = 0.586	Dev. Explained = 62.7 %		

Table S5 Summary results from Generalized Additive Models with log-transformed concentrations of trace elements as response variables, considering bird blood samples collected in the marine offshore habitat (Trindade Island), Brazil. Significant p-values are presented in bold.

**Generalized Additive Models – Offshore**				
**Lead (Pb)**	Effect. Df	Res. Df	F	**p-value**
$\delta^{13}C$	0	9	0	0.938
$\delta^{15}N$	1.1	9	0.222	0.156
$\delta^{34}S$	2.87	9	1.548	**0.002**
	R2 = 0.655	Dev. Explained = 68.8%		
**Cadmium (Cd)**	Effect. Df	Res. Df	F	**p-value**
$\delta^{13}C$	1.43	9	0.39	0.079
$\delta^{15}N$	0	9	0	0.909
$\delta^{34}S$	0	9	0	0.901
	R2 = 0.0027	Dev. Explained = 6.8%		
**Arsenic (As)**	Effect. Df	Res. Df	F	**p-value**
$\delta^{13}C$	0	9	0	0.373
$\delta^{15}N$	0	9	0	0.336
$\delta^{34}S$	0.79	9	0.423	**0.031**
	R2 = 0.672	Dev. Explained = 69.1%		
**Mercury (Hg)**	Effect. Df	Res. Df	F	**p-value**
$\delta^{13}C$	0	9	0	0.728
$\delta^{15}N$	0.99	9	0.235	0.116
$\delta^{34}S$	0.35	9	0.054	0.236
	R2 = 0.699	Dev. Explained = 71.8%		

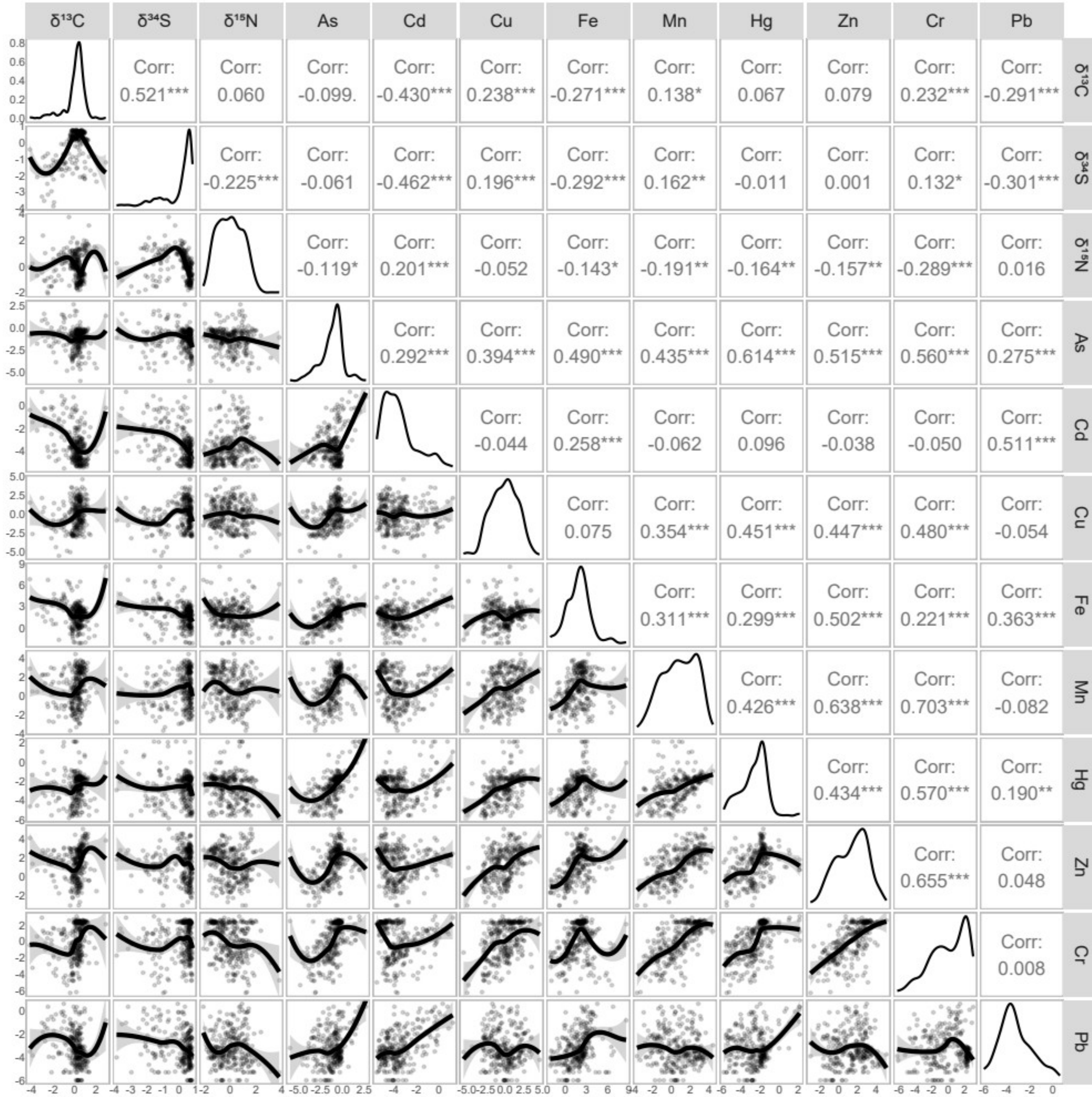

Fig. S1 Pearson correlation between scaled isotope values and log-transformed trace element concentrations in bird blood, collected across estuarine, nearshore and offshore habitats in Brazil. * $p < 0.05$, ** $p < 0.01$, *** $p < 0.001$.

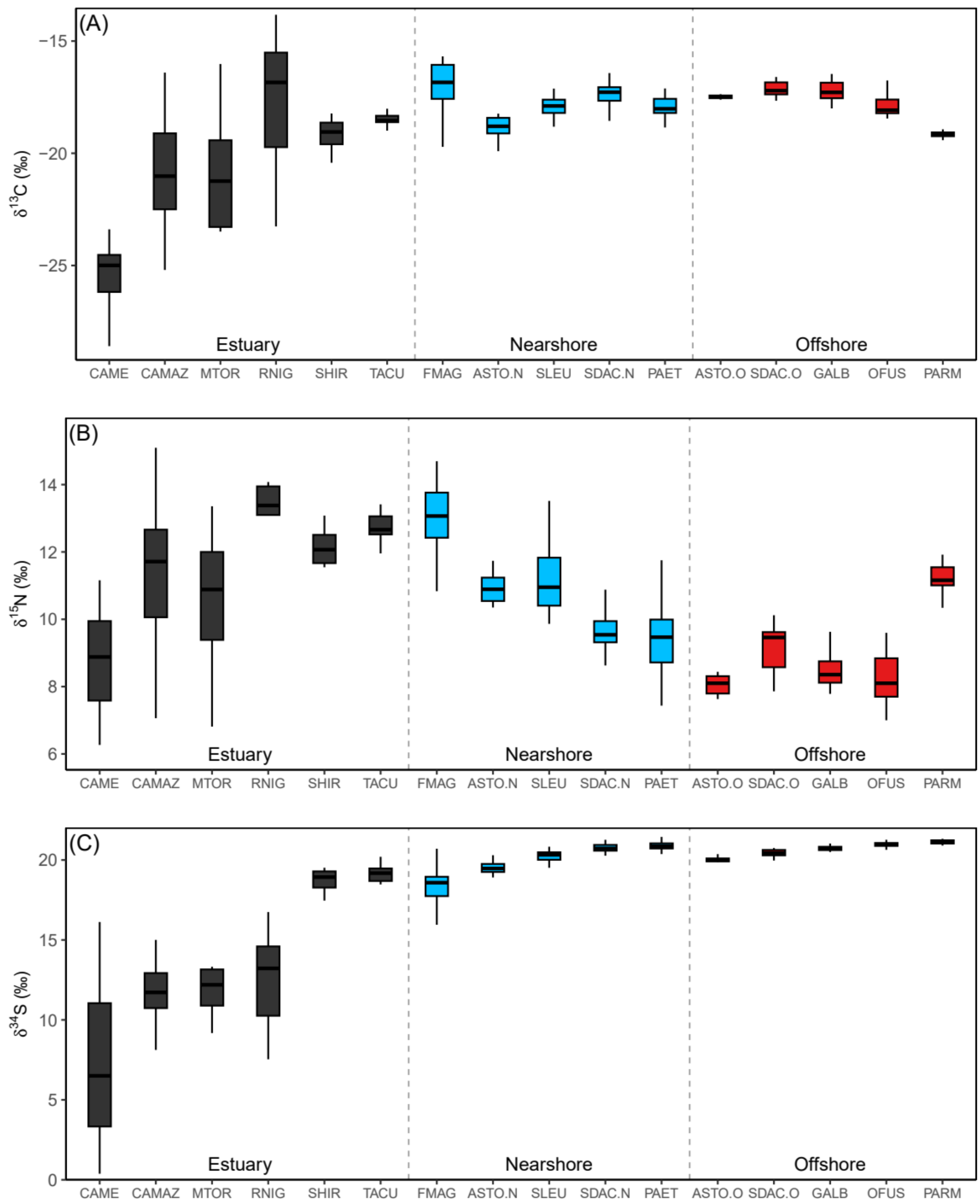

Fig. S2. Variation in stable carbon (A), nitrogen (B) and sulfur (C) isotope values in the blood of piscivorous bird species sampled across estuarine (dark grey), nearshore (blue) and offshore (red) habitats in southeast Brazil and the southwestern Atlantic Ocean. Boxes indicate medians and interquartile ranges. Abbreviations: CAME = *Chloroceryle americana*; CAMAZ = *C. amazona*; MTOR

= *Megaceryle torquata*; RNIG = *Rynchops niger*; SHIR = *Sterna hirundo*; TACU = *Thalasseus acuflavidus*; ASTO = *Anous stolidus*; PAET = *Phaethon aethereus*; SDAC = *Sula dactylatra*; SLEU = *S. leucogaster*; FMAG = *Fregata magnificens*; OFUS = *Onychoprion fuscatus*; GALB = *Gygis alba*; and PARM = *Pterodroma arminjoniana*. Abbreviations of species sampled in nearshore and offshore habitats are followed by the first letter of the habitat after a period ('.N' and '.O', respectively).

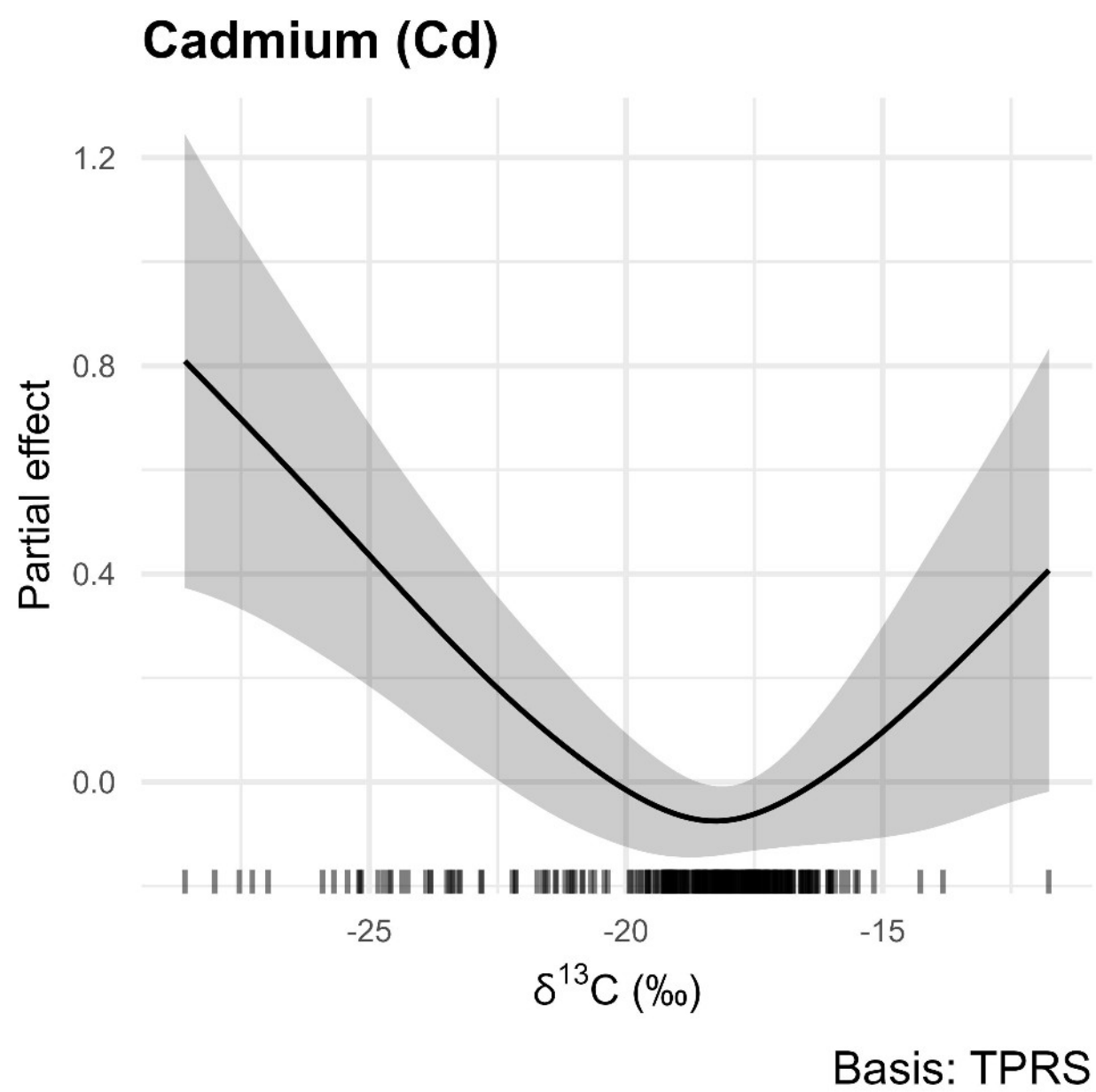

Fig. S3. Partial effect of δ^{13}C values (in ‰) over cadmium (Cd) measured in bird blood samples collected in estuarine, nearshore and offshore habitats in Brazil, as estimated from Generalized Additive Models (GAMs).

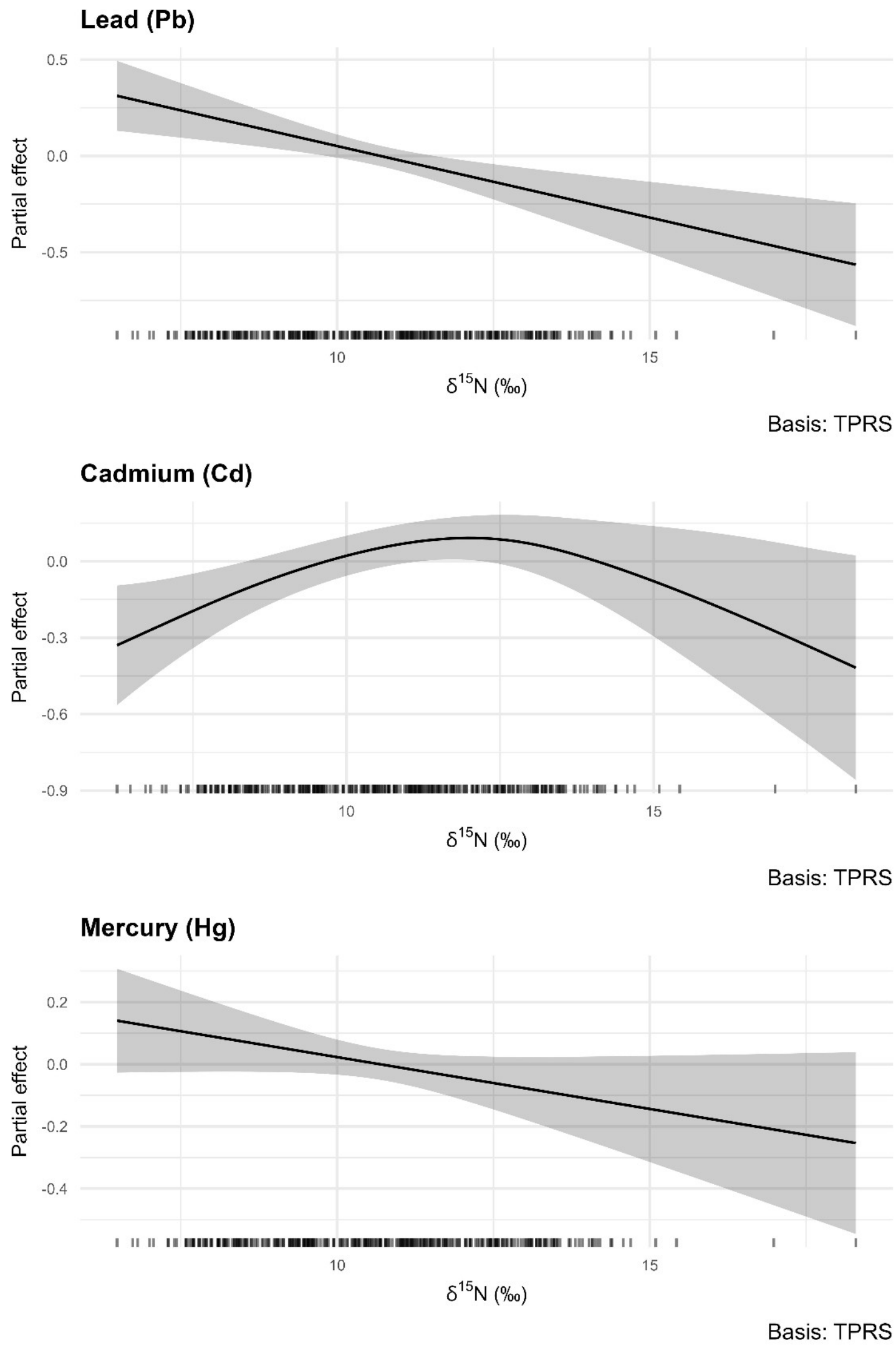

Fig. S4. Smooths obtained with Generalized Additive Models (GAMs), representing the partial effect of nitrogen isotope values (δ^{15}N) over (log-transformed) non-essential trace elements measured in bird blood samples collected in estuaries, nearshore (Abrolhos Archipelago) and offshore (Trindade Island) marine habitats in Brazil.

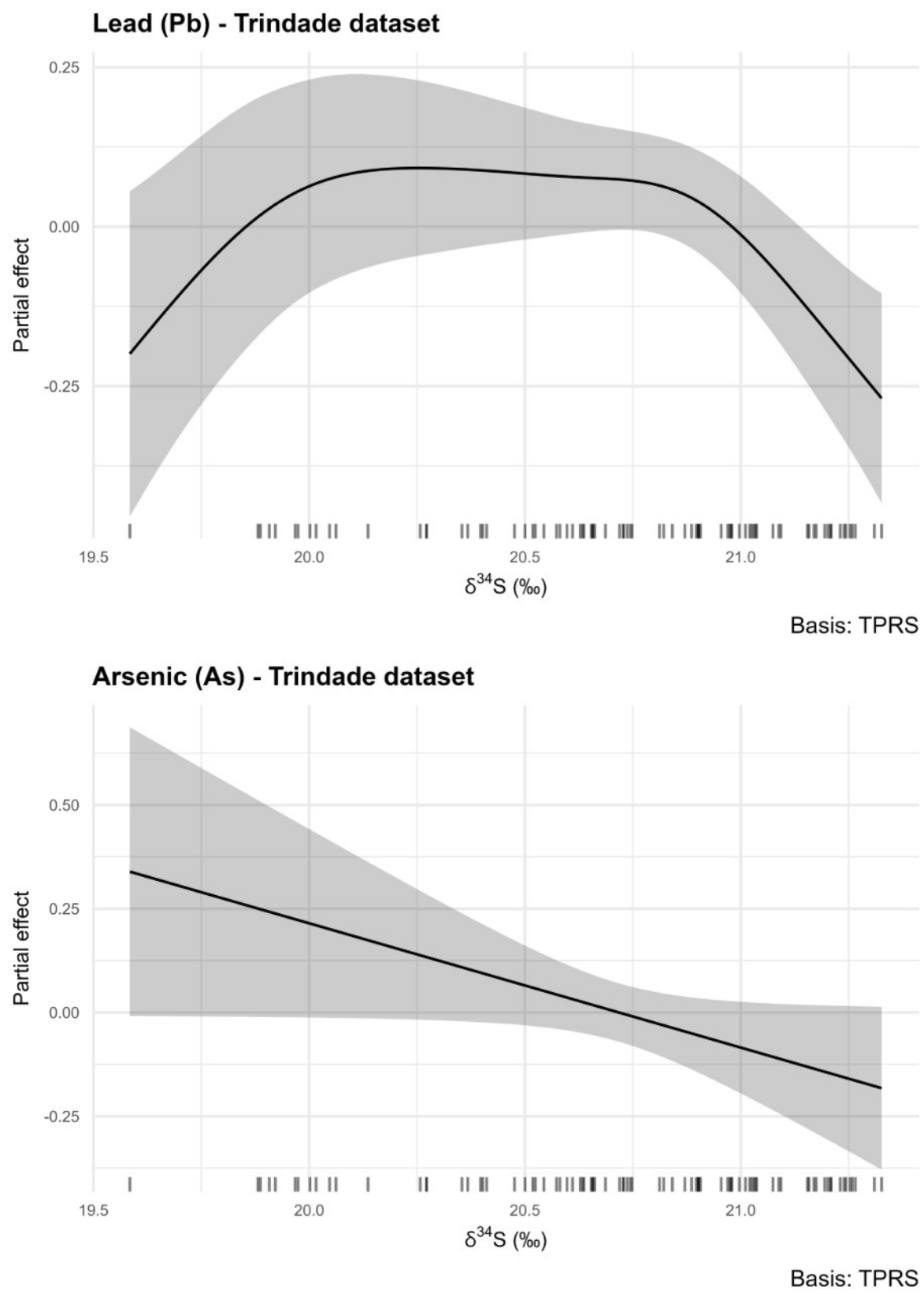

Fig. S5. Partial effect smooths of $\delta^{34}S$ over non-essential trace elements analyzed in the blood of birds sampled in the offshore Trindade Island in Brazil, as obtained through Generalized Additive Models.
